# Modeling net ecosystem carbon balance and loss in coastal wetlands exposed to sea‐level rise and saltwater intrusion

**DOI:** 10.1002/eap.2702

**Published:** 2022-08-12

**Authors:** Khandker S. Ishtiaq, Tiffany G. Troxler, Lukas Lamb‐Wotton, Benjamin J. Wilson, Sean P. Charles, Stephen E. Davis, John S. Kominoski, David T. Rudnick, Fred H. Sklar

**Affiliations:** ^1^ Sea Level Solutions Center, Institute of Environment Florida International University Miami Florida USA; ^2^ Department of Earth and Environment Florida International University Miami Florida USA; ^3^ Department of Biological Sciences Florida International University Miami Florida USA; ^4^ Texas General Land Office Austin Texas USA; ^5^ Department of Coastal Studies East Carolina University Wanchese North Carolina USA; ^6^ Everglades Foundation Palmetto Bay Florida USA; ^7^ Everglades National Park Homestead Florida USA; ^8^ South Florida Water Management District West Palm Beach Florida USA

**Keywords:** elevation change, net ecosystem C balance, peat collapse, saltwater intrusion, sea‐level rise, wetland vulnerability

## Abstract

Coastal wetlands are globally important stores of carbon (C). However, accelerated sea‐level rise (SLR), increased saltwater intrusion, and modified freshwater discharge can contribute to the collapse of peat marshes, converting coastal peatlands into open water. Applying results from multiple experiments from sawgrass (*Cladium jamaicense*)‐dominated freshwater and brackish water marshes in the Florida Coastal Everglades, we developed a system‐level mechanistic peat elevation model (EvPEM). We applied the model to simulate net ecosystem C balance (NECB) and peat elevation in response to elevated salinity under inundation and drought exposure. Using a mass C balance approach, we estimated net gain in C and corresponding export of aquatic fluxes ($F_{\mathrm{AQ}}$) in the freshwater marsh under ambient conditions (NECB = 1119 ± 229 gC m^−2^ year^−1^; *F*_AQ_ = 317 ± 186 gC m^−2^ year^−1^). In contrast, the brackish water marsh exhibited substantial peat loss and aquatic C export with ambient (NECB = −366 ± 15 gC m^−2^ year^−1^; *F*_AQ_ = 311 ± 30 gC m^−2^ year^−1^) and elevated salinity (NECB = −594 ± 94 gC m^−2^ year^−1^; *F*_AQ_ = 729 ± 142 gC m^−2^ year^−1^) under extended exposed conditions. Further, mass balance suggests a considerable decline in soil C and corresponding elevation loss with elevated salinity and seasonal dry‐down. Applying EvPEM, we developed critical marsh net primary productivity (NPP) thresholds as a function of salinity to simulate accumulating, steady‐state, and collapsing peat elevations. The optimization showed that ~150–1070 gC m^−2^ year^−1^ NPP could support a stable peat elevation (elevation change ≈ SLR), with the corresponding salinity ranging from 1 to 20 ppt under increasing inundation levels. The C budgeting and modeling illustrate the impacts of saltwater intrusion, inundation, and seasonal dry‐down and reduce uncertainties in understanding the fate of coastal peat wetlands with SLR and freshwater restoration. The modeling results provide management targets for hydrologic restoration based on the ecological conditions needed to reduce the vulnerability of the Everglades' peat marshes to collapse. The approach can be extended to other coastal peatlands to quantify C loss and improve understanding of the influence of the biological controls on wetland C storage changes for coastal management.

## INTRODUCTION

Coastal wetlands, one of the most valuable reservoirs of blue carbon (C) (Mcleod et al., 2011; Windham‐Myers et al., 2018), are encountering a shift in C sink capacity, vulnerability to submergence, and transformation of ecological functionality due to accelerated sea‐level rise (SLR) and saltwater intrusion (Lovelock et al., 2015; Rodriguez et al., 2017; Rogers et al., 2019). In a number of coastal peatlands, SLR‐driven hydrologic and biogeochemical stresses are drastically altering the marsh community and subsequently reshaping the ability of marsh ecosystems to maintain elevation to keep pace with SLR (Morris et al., 2002; Neubauer et al., 2013). Sudden elevation loss in organic peat soils is referred to as peat collapse and results in the subsequent conversion of vegetated peat marsh to open water (Chambers et al., 2019; Cooper & Zhang, 2020; Wilson et al., 2018). In the Florida Coastal Everglades, peat collapse is connected to a shifting hydroperiod due to hydrologic interventions and decline in marsh productivity due to increasing salinity (Charles et al., 2019; Wilson et al., 2018). Because of the far‐reaching adverse consequences of peat collapse in coastal wetlands, estimation of net C change in response to changing inundation and salinity can provide improved assessments of the vulnerability of coastal peatlands to SLR.

Mechanisms for peat collapse involve a multitude of biological and physical processes. In general, the total elevation loss (i.e., subsidence) in peat soil is the sum of peat compression due to compaction/consolidation and organic matter oxidation (Kool et al., 2006). The biological process of peat loss involves a change in ecosystem C balance and corresponding changes in productivity and decomposition, which are strongly mediated by hydrologic and salinity stresses in coastal systems. With ambient salinity and moderate submergence, ecosystem productivity tends to be higher than decomposition, resulting in a positive C balance. Although excessive flooding can cause a reduction in plant productivity and higher mortality rates (Kirwan & Megonigal, 2013; Troxler et al., 2014), elevated salinity is known to reduce photosynthetic efficiency, growth rates, root productivity, and nutrient uptake (Charles et al., 2019; Rejmankova & Macek, 2008; Richards & Olivas, 2020; Solohin et al., 2020). Persistent or seasonal dry‐down contributes to release of C to the atmosphere in the form of CO_2_ and increases the rate of elevation loss because of soil oxidation, which is also negatively influenced by changing salinity (Chambers et al., 2014; Khasanah & van Noordwijk, 2019; Wang et al., 2015). The physical process of peat elevation (PE) loss includes drainage or soil disturbance that can cause higher compaction in peat soil; however, the degree of compaction has been found to decrease with time as soil bulk density increases (Aich et al., 2013; Kool et al., 2006). Based on a synthesis of experimental work and observations, Chambers et al. (2019) linked peat collapse to compaction of soil pore space during drought, deconsolidation of submerged peats, degradation of roots, and soil mineralization.

Peat collapse in coastal wetlands has been evident around the United States, particularly in the interior zones that have limited or no supply of mineral sediments (Chambers et al., 2019; DeLaune et al., 1994). An ample portion of Louisiana marshes continues to undergo peat loss due to SLR and saltwater intrusion amid an inadequate supply of mineral sediments (Day et al., 2011; DeLaune et al., 1994; Sapkota & White, 2019). Hydrologic modifications and other disturbances have resulted in the conversion of marsh ecosystems to open water in the Cape Sable area of Everglades National Park in Florida, where ~2–4 m of peat was lost (Wanless & Vlaswinkel, 2005). The Florida Everglades, the largest subtropical wetland in the United States, is currently experiencing saltwater intrusion from a combination of SLR and freshwater diversion (Dessu et al., 2018; Pearlstine et al., 2010). Over the past 100 years, the Everglades has endured enormous hydrologic modifications through the creation of canals and pumps to divert freshwater flows, resulting in annual dry‐down and gradual saltwater intrusion. At the interface between the Everglades' freshwater sawgrass marshes and mangrove ecosystems, sawgrass marshes are increasingly exposed to brackish water, making them vulnerable to peat soil degradation due to changes in plant productivity and decomposition that maintain peat soil elevation (Wilson, 2018; Wilson et al., 2018).

Although efforts have been made to increase upstream freshwater flow to counterbalance saltwater intrusion through Everglades restoration activities (Sklar et al., 2005; Wetzel et al., 2017), projected SLR of 2–2.5 m by the end of this century will continue to alter hydrological and ecological regimes through inundation and saltwater intrusion (Sweet et al., 2017). For instance, different landscape‐scale hydrobiogeochemical models predict an increase of inundation and saltwater intrusion within the coastal Everglades (Flower et al., 2017) amid a considerably higher relative SLR, particularly in sawgrass marshes (Meeder et al., 2017). By balancing ecosystem productivity and loss rates, Troxler et al. (2013) estimated net ecosystem C balance (NECB) for different vegetation communities of the Everglades that provided estimates of C accumulation and lateral transport of aquatic C flux. However, potential changes in NECB because of SLR and increasing salinity in the Florida Coastal Everglades remain unquantified. Therefore, direct and model‐based estimations of the marsh C budget and subsequent elevation changes in response to SLR, saltwater intrusion, and seasonal dry‐down are important to support comprehensive wetland restoration efforts.

In recent years, several experimental studies have been carried out in freshwater and brackish water sawgrass marshes in the Florida Coastal Everglades to understand how salinity and changing inundation depths (flooding/exposure) interact to drive C balance and PE loss (Charles et al., 2019; Servais et al., 2020; Wilson et al., 2018, 2019). Based on these experimental manipulations, Wilson et al. (2018) reported a decline in brackish water sawgrass marsh productivity and C loss with elevated salinity under flooding and exposed conditions. Charles et al. (2019) found that leaf and root breakdown rates increased with elevated salinity along with a decrease in belowground biomass, resulting in soil C loss in brackish water marshes for both periodically dried and inundated hydrologic treatments. Servais et al. (2020) reported an enhanced root litter decomposition rate and a corresponding loss of soil C based on plant–soil monoliths from the brackish water marsh. These studies have provided experimentally derived data sets for freshwater and brackish water sawgrass peat marshes under ambient and elevated salinity levels with submerged, exposed, and extended‐exposed peat surfaces.

Despite the growing observational and experimental evidence of peat collapse, a limited number of studies have applied models to simulate C balance and soil elevation that incorporates the biological control of peat collapse (Alizad et al., 2016; Braun et al., 2019; Reed et al., 2020; White et al., 2019). Alizad et al. (2016) developed the Hydro‐MEM model to evaluate the impacts of SLR on North Florida salt marshes by integrating a spatially explicit hydrodynamic model and an ecological marsh equilibrium model (MEM; Morris et al., 2002). Reed et al. (2020) utilized the wetland morphology change model (ICM‐Morph) to examine the spatial and temporal variability of Louisiana wetland loss and reported that saltwater intrusion is the most critical factor accounting for wetland loss, although the model did not directly include the influence of elevated salinity or modified hydrology on vegetation productivity and decomposition rates.

**FIGURE 1:**
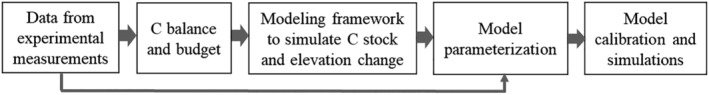
Work flow diagram illustrating how the collected experimental data at varying levels of salinity and inundations were utilized to compute net ecosystem carbon balance and develop mechanistic modeling framework for parameterization and simulations of peat stock and peat elevation change.

Freshwater restoration is expected to preserve peat and reduce wetland vulnerability (NASEM, 2018; Sklar et al., 2005), yet the impact of salinity across the landscape has not been systematically quantified. The overall goal of this study was to understand changes in C balance and elevation in coastal peatlands with elevated salinity and hydrologic alterations that would help to better comprehend coastal vulnerability and inform management targets for hydrologic restoration. We addressed two underlying research questions. First, how much net C will coastal peatlands release due to saltwater intrusion and hydrologic modifications? Second, what is the estimated marsh plant production (sawgrass) necessary to maintain and enhance peat accretion under saltwater intrusion and SLR? To address these questions, we estimated NECB and completed C budget(s) in the Everglades marshes using measured experimental data (biomass, productivity, decomposition rates, net ecosystem exchange [NEE] of CO_2_ and CH_4_, and elevation change) from a freshwater and a brackish water sawgrass (*Cladium jamaicense*) peat marsh. We used this information to develop a mechanistic model to simulate the wetland soil C balance and elevation change in response to inundation and salinity (Figure 1). We synthesized the experimental data (Charles et al., 2019; Servais et al., 2020; Wilson, 2018; Wilson et al., 2018, 2019) representing sawgrass‐dominated freshwater and brackish water marshes to empirically parameterize the associated model equations. We used a system dynamics modeling tool (Stella, Isee System, 2006) to develop and calibrate the model. Based on the model simulation, we evaluated different critical primary productivity thresholds as a function of salinity for accumulating, steady‐state, and collapsing peats under increasing levels of inundation. The developed model is a novel tool for quantifying peat C balance and elevation change as a function of primary productivity (above‐ and belowground), water level (WL), and porewater salinity. The model can be applied to (a) assess peat collapse vulnerability, (b) simulate future scenarios with changing productivity, hydroperiod, and salinity, and (c) derive wetland restoration targets under ongoing SLR and saltwater intrusion. Furthermore, as the comprehensive Everglades restoration plan involves restoring more natural wetland hydropatterns and minimizing the rate and extent of saltwater intrusion through upstream water management to minimize ecosystem degradation, the developed model and estimated C budget can inform restoration and management practices to reduce the peat collapse vulnerability of the Florida Everglades in the face of accelerated SLR.

## MATERIALS AND METHODS

### Study sites

We assembled experimental data from a freshwater peat marsh and a brackish water peat marsh located within the Florida Everglades to estimate and simulate C balance and PE change in response to saltwater intrusion and level of inundation. The freshwater site (25°26′07.38″ N, 80°46′50.71″ W) is a nontidal peat marsh that is subjected to a long‐hydroperiod regime and remains flooded for almost 11–12 months in a year. The ambient soil bulk density ($\rho_{b}$) of the freshwater soil was 0.08 ± 0.03 g cm^−3^ and the soil C content ($f_{C}$) was 41% within the top 10 cm (Wilson, 2018; Wilson et al., 2018). The porewater salinity of the site was <0.5 ppt. In contrast, the nontidal brackish water peat marsh (25°13′13.74″ N, 80°50′36.81″ W) is a relatively short‐hydroperiod marsh (about 8–10 months of flooding) that has experienced a gradual increase in salinity over time due to SLR while also visibly experiencing peat collapse, indicated by the presence of peat pedestals (Wilson et al., 2018). The reported $\rho_{b}$ and $f_{C}$ within the top 10 cm of soil in the brackish water site were 0.13 ± 0.01 g cm^−3^ and 43%, respectively (Wilson et al., 2018). The mean ambient porewater salinity of the site was around 9 to 10 ppt during the data collection phase. The freshwater and brackish water marsh experimental sites were dominated by *C. jamaicense* (sawgrass), with *Conocarpus erectus* (buttonwood) and *Rhizophora mangle* (red mangrove) also present at the brackish water site. The depth of the peat soil is ~0.5 and 1.5–2.0 m overlying a limestone bedrock in the freshwater and brackish water marsh sites, respectively.

### Description of experiments for C budgeting and modeling

The data were collected from multiple outdoor laboratory mesocosm experiments conducted at the Florida Bay Interagency Science Center in Key Largo, FL, during 2015–2017 (Charles et al., 2019; Servais et al., 2020; Troxler et al., 2022; Wilson, 2018; Wilson et al., 2018, 2019) using sawgrass plant‐peat monoliths from the freshwater and brackish water marsh sites. In the experiment that focused on the freshwater marsh, six monoliths were used; the plots were kept inundated under ~1 cm of water relative to the surface and ambient porewater salinity (~0.5 ppt) for 1 year. In experiments focused on the brackish water marsh, three experiments were carried out reflecting the combined effect of salinity, inundation, and peat exposure to air. The brackish water experiments were characterized by submerged (SUB), exposed (EXP), and extended depth of exposure of peat surface (EXTEXP) conditions, as we varied water depth relative to the peat surface. Each experiment was subjected to two salinity manipulations: (1) ambient (~10 ppt) porewater salinity (AMB) and (2) elevated (~20 ppt) salinity (SALT). The experimental design included six (2 × 3) treatments: (1) submerged ambient salinity (AMB.SUB), (2) submerged elevated salinity (SALT.SUB.), (3) exposed ambient salinity (AMB.EXP), (4) exposed elevated salinity (SALT.EXP), (5) exposed with extended exposure/dry‐down ambient salinity (AMB.EXTEXP), and (6) exposed with extended exposure/dry‐down elevated salinity (SALT.EXTEXP). The WL was kept 4 cm above the peat surface for the brackish water SUB treatments. Exposure for the EXP treatment was 4 cm below the soil surface. In EXTEXP treatment, the exposure of the peat surface varied from 0 to 20 cm for ~6 months in order to simulate drought, where the length of the dry season was extended, relative to field conditions at the brackish water marsh (Appendix S1: Figure S1). From these experiments, data for productivity and litter decomposition, soil physical properties, surface–atmosphere CO_2_ exchange, and change in PE were used to estimate C balance and parameterize and develop the Everglades peat elevation model.

### Measurements and data sets

A detailed description of the measurements and processes of productivity, decomposition rates, net exchanges of CO_2_ and CH_4_, and PE changes of the treatments applied can be found in several sources (Charles et al., 2019; Servais et al., 2020; Wilson, 2018; Wilson et al., 2018, 2019).

Bimonthly measurements of plant height, culm diameter, and stem density were used to calculate the aboveground net primary productivity (ANPP; Childers et al., 2006). Cylindrical mesh bags were used to estimate the root productivity (belowground net primary productivity [BNPP]; Vogt et al., 1998). We estimated the aboveground and belowground turnover rates from the ratio between average productivity and biomass (Castaneda‐Moya et al., 2011; Eissenstat & Yanai, 1997). The leaf ($k_{\mathrm{AG}}$) and root ($k_{\mathrm{BG}}$) litter decomposition rates were estimated by regressing the proportion of ash‐free dry mass (AFDM) remaining with time assuming an exponential function for all but the extended‐exposed treatments (Charles et al., 2019). We did not use exponential regression to estimate the decomposition rates for the EXTEXP treatments because only the final remaining mass of the organic soil in the litterbag was measured without the intermittent fractional time steps. As an alternative, leaf and root decomposition rates ($k_{\mathrm{AG}}$ and $k_{\mathrm{BG}}$) for the EXTEXP treatments were calculated from the ratio between litter decomposition and production, where litter production was estimated by multiplying the biomass by the corresponding turnover rates. We calculated the degree of compaction, α as the percentage change of $\rho_{b}\;$due to experiments from baseline field $\rho_{b}$ for the brackish water and freshwater peats. To measure the net ecosystem exchange of CO_2_ (NEE) for each plant–soil monolith, a sealed transparent chamber was used. We measured the soil methane (CH_4_) fluxes from the monoliths for the freshwater, brackish water AMB.EXP, and SALT.EXP treatments; however, CH_4_ fluxes were not measured for the brackish water EXTEXP treatments. PE was measured 1 year after the start of the experiments from a fixed benchmark relative to the baseline peat surface. From the full PE data set, we omitted three of six experimental units from the freshwater experiment due to errant values we attributed to an experimental artifact or measurement error. Our rationale was based on long‐term field observations and radiocarbon dating (e.g., Saunders et al., 2007; Troxler et al., 2013) that an Everglades freshwater peat marsh under ambient conditions accumulates C and gains elevation in a system with high primary productivity and high CO_2_ uptake rates.

### Estimating net ecosystem C balance

**FIGURE 2:**
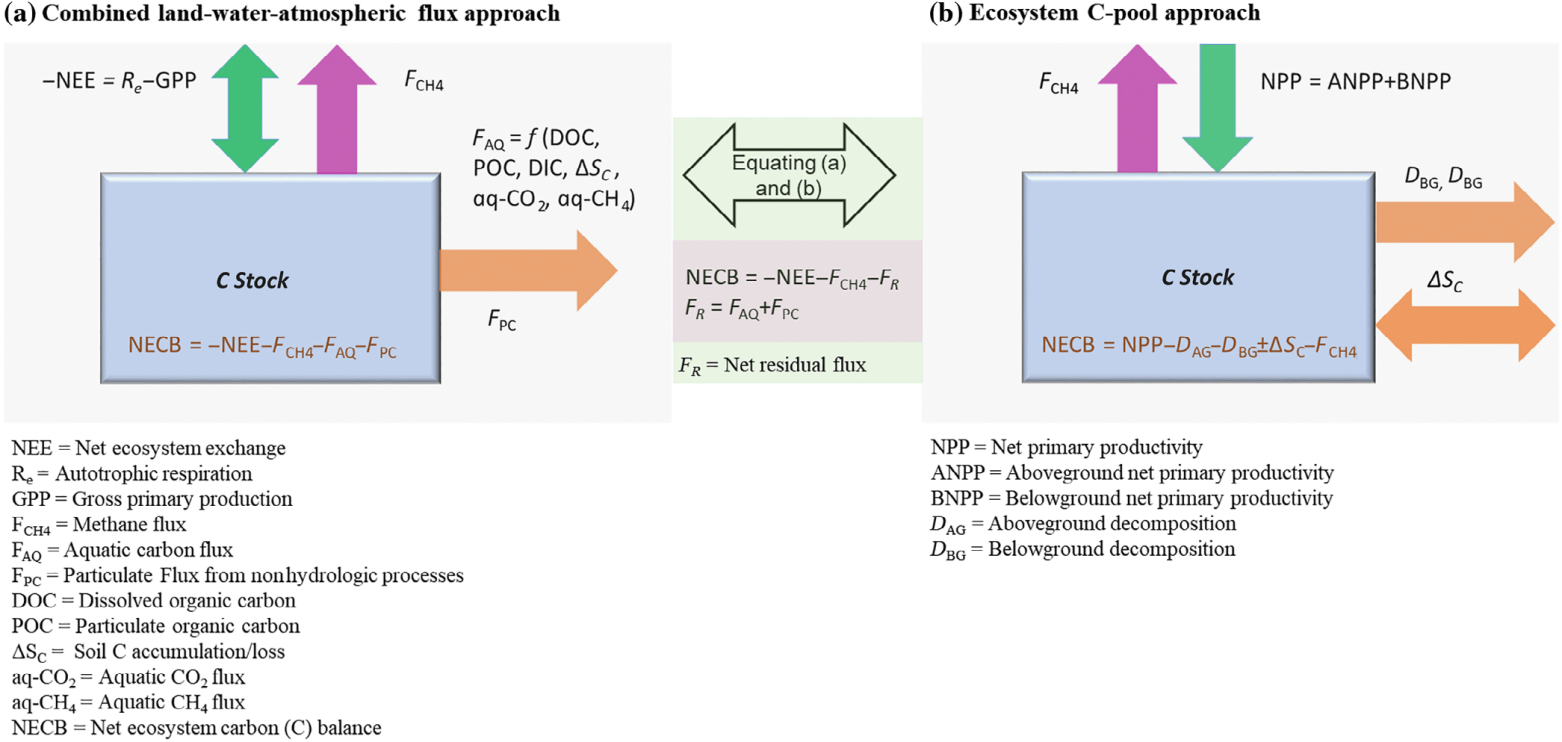
Conceptual schematic showing balancing of ecosystem carbon (C) budget. The net residual C flux (*F*_*R*_) is estimated by equating (a) land–water–atmospheric flux and (b) ecosystem C pool–based C balance approaches. *F*_*R*_ represents remaining components of C budget such as the fraction of *F*_AQ_ (e.g., dissolved inorganic carbon, dissolved organic carbon, particulate organic carbon), and *F*_PC_ that are not incorporated in approach (b).

The NECB represents the net change in C within the ecosystem and can be defined by the algebraic sum of the input and outputs from all process pathways, including net vertical ecosystem exchanges of CO_2_ and CH_4_ fluxes and lateral exchanges of aquatic and particulate C fluxes (Figure 2a; Chapin et al., 2006; Webb et al., 2019). Following the concept of ecosystem mass balance, Webb et al. (2019) approximated the ecosystem C budget as follows (Equation 1):

(1) $$\text{NECB}=-\mathrm{NEE}-F_{\mathrm{CH}4}-\left(F_{\mathrm{AQ}}+F_{\mathrm{PC}}\right)$$

where NEE and $F_{\mathrm{CH}4}$ respectively refer to net ecosystem exchange of CO_2_ and CH_4_. A positive sign of NEE and $F_{\mathrm{CH}4}$ indicates net loss to the atmosphere from the ecosystem. $F_{\mathrm{AQ}}$ represents hydrologic discharge‐driven lateral exchanges of total aquatic C fluxes consisting of dissolved organic carbon (DOC), dissolved inorganic carbon (DIC), particulate organic carbon (POC), gaseous exchange of CO_2_ and CH_4_, and soil C accumulation or loss via hydrologic runoff. $F_{\mathrm{PC}}$ refers to lateral exchanges of particulate C originated from soot emission, loss (erosion) and accumulation (deposition) due to disturbances that are not related to hydrologic discharge (e.g., wind flow), and animal movement. A positive $F_{\mathrm{AQ}}$ and $F_{\mathrm{PC}}$ refer to the net export of C, and a positive sign of NECB indicates net accumulation within the ecosystem.

The net C balance, NECB, can also be estimated based on the ecosystem C‐pool approach using a mass‐balance equation (Equation 2, Figure 2b; Lovett et al., 2006; Troxler et al., 2013):

(2) $$\text{NECB}=\text{ANPP}+\text{BNPP}+D_{\mathrm{AG}}+D_{\mathrm{BG}}+∆S_{C}-F_{\mathrm{CH}4}$$

where $D_{\mathrm{AG}}$, $D_{\mathrm{BG}}$, and $∆S_{C}$ refer to aboveground leaf decomposition, belowground root decomposition, and change in soil C, respectively. A positive sign of ANPP and BNPP indicates net input to the system, while a negative sign of $D_{\mathrm{AG}}$, $D_{\mathrm{BG}}$, and $∆S_{C}$ represents net loss. $D_{\mathrm{AG}}$ and$\;D_{\mathrm{BG}}$ include heterotrophic respiration as well as components of $F_{\mathrm{AQ}}$ (e.g., DOC). $∆S_{C}$, which is also a component of $F_{\mathrm{AQ}}$ and $F_{\mathrm{PC}}$, represents a change in soil C due to organic accumulation/loss and leaching/export. Further, ANPP and BNPP in Equation (2) combinedly represent the net primary productivity (NPP).

Replacing C stock‐based estimates of NECB in Equation (1), we can compute the net residual C flux ($F_{R}$) that represents the remaining components of the budget (Equations 3 and 4) (Figure 2):

(3) $$F_{R}=F_{\mathrm{AQ}}+F_{\mathrm{PC}}$$

(4) $$\text{NECB}=-\mathrm{NEE}-F_{\mathrm{CH}4}-F_{R}$$

where a positive $F_{R}$ represents net export and consists of the missing components such as $F_{\mathrm{PC}}$ and fraction of $F_{\mathrm{AQ}}$ (e.g., DIC, DOC, POC) those are not accounted for in Equation (2). Assuming negligible $F_{\mathrm{PC}}$ in Equation (4), $F_{\mathrm{AQ}}$ can be approximated as a sum of $F_{R}\;$ and $-∆S_{C}$ (Equation 5):

(5) $$F_{\mathrm{AQ}}=F_{R}-∆S_{C}$$

The $∆S_{C}$ in Equation (2) was estimated from the total soil mass ($∆S_{T})$, considering the fraction of C in the soil matter ($f_{C}$), change in peat depth (∆PD) and degree of compaction (i.e., compression other than material loss), α (Equations 6 and 7) (Khasanah & van Noordwijk, 2019). $∆S_{T}$ represents the net change in soil from processes other than compaction/swelling and aboveground/belowground decompositions. Bulk density of organic soil, $\rho_{b}$, was used as a multiplication factor to convert to flux units. Further, we subtracted $D_{\mathrm{AG}}$ and $D_{\mathrm{BG}}$ while calculating $∆S_{T}$ to avoid double counting total mass exchanges:

(6) $$∆S_{C}=∆S_{T}\times f_{C}$$

(7) $$∆S_{T}=\left(1-\alpha \right)∆\mathrm{PD}\times \rho_{b}-\left(D_{\mathrm{AG}}+D_{\mathrm{BG}}\right)$$

where the value of α ranged from 0 to 1, where 1 represents 100% compaction of soil.

### Modeling framework

We present a system‐level dynamic mechanistic modeling framework to simulate variation in peat soil stock (depth of soil over the bed rock), $M_{S}$ (Equation 8), as a function of salinity and inundation by balancing the total inputs and outputs of mass across the system at a daily time scale:

(8) $$M_{S}\left(t\right)=M_{S}\;\left(t-1\right)+C_{\mathrm{AG}}\left(t\right)+C_{\mathrm{BG}}\left(t\right)+∆S_{T}\left(t\right)-F_{\mathrm{CH}4}\left(t\right)$$

where $C_{\mathrm{AG}}$ and $C_{\mathrm{BG}}$ respectively refer to the aboveground and belowground contribution to the peat soil stock for a time t, and $-F_{\mathrm{CH}4}$ represents loss from $M_{S}$. $M_{S}\left(t-1\right)$ represents the soil stock in the previous time step of t−1. $C_{\mathrm{AG}}$, $C_{\mathrm{BG}}$, and $-F_{\mathrm{CH}4}$ are in units of gC m^−2^ day^−1^, while the unit of $M_{S}$ and $∆S_{T}$ is g m^−2^ day^−1^. For the initial condition, $M_{S}\left(t_{0}\right)\;$is the product of the initial depth of peat soil above the bedrock, $h_{0}$, and soil bulk density, $\rho_{b}$ (Equation 9)

(9) $$M_{S}\left(t_{0}\right)=h_{0}\times \rho_{b}$$

where $h_{0}$ is in m and $\rho_{b}$ is in g m^−3^. The simulated soil stock, $M_{S}\left(t\right)$ in Equation (8), is then converted to equivalent peat depth (${\mathrm{PD}}_{e}$) from $\rho_{b}$ in each time step t (Equation 10):

(10) $${\mathrm{PD}}_{e}\left(t\right)=\frac{M_{S}\left(t\right)}{\rho_{b}}$$

${\mathrm{PD}}_{e}$ is then adjusted for soil compaction based on the degree of compaction, $\alpha \left(t\right)$, to estimate the total change in peat depth (${\mathrm{PD}}_{a})$ (Equation 11). The input α can vary with time to incorporate the impact of initial compaction and long‐term relatively slow consolidation:

(11) $${\mathrm{PD}}_{a}\left(t\right)={\mathrm{PD}}_{e}\left(t\right)-\frac{\alpha \;\left(t\right)}{1-\alpha \left(t\right)}∆{\mathrm{PD}}_{e}$$

where ${\mathrm{PD}}_{a}$ is in meters, which is converted to centimeters, and then transferred to PE relative to a known elevation benchmark (EB) in centimeters of the North American Vertical Datum of 1988 (NAVD 88) using Equation (12):

(12) $$\mathrm{PE}\left(t\right)=\mathrm{PD}\left(t\right)\times 100+\mathrm{EB}$$

$C_{\mathrm{AG}}$ and $C_{\mathrm{BG}}$ in Equation (8) are determined based on the litter production and decomposition for both aboveground and belowground C (Equations 13 and 14):

(13) $$C_{\mathrm{AG}}\left(t\right)=\left\{\begin{array}{cc} {\mathrm{LP}}_{\mathrm{AG}}\left(t\right)-D_{\mathrm{AG}}\left(t\right), & \text{when}\;{\mathrm{LP}}_{\mathrm{AG}}\left(t\right)>D_{\mathrm{AG}}\left(t\right)\\ 0, & \text{when}\;{\mathrm{LP}}_{\mathrm{AG}}\left(t\right)\leq D_{\mathrm{AG}}\left(t\right) \end{array}\right.$$

(14) $$C_{\mathrm{BG}}\left(t\right)=\left\{\begin{array}{cc} {\mathrm{LP}}_{\mathrm{BG}}\left(t\right)-D_{\mathrm{BG}}\left(t\right), & \text{when}\;{\mathrm{LP}}_{\mathrm{BG}}\left(t\right)>D_{\mathrm{BG}}\left(t\right)\\ 0, & \text{when}\;{\mathrm{LP}}_{\mathrm{BG}}\left(t\right)\leq D_{\mathrm{BG}}\left(t\right) \end{array}\right.$$

where ${\mathrm{LP}}_{\mathrm{AG}}$ and ${\mathrm{LP}}_{\mathrm{BG}}$ respectively refer to aboveground leaf litter production and belowground root litter production in gC m^−2^ day^−1^. ${\mathrm{LP}}_{\mathrm{AG}}\;\text{and}$ ${\mathrm{LP}}_{\mathrm{BG}}$ respectively contribute to the aboveground (${\mathrm{LS}}_{\mathrm{AG}}$) and belowground (${\mathrm{LS}}_{\mathrm{BG}}$) litter stocks. ${\mathrm{LS}}_{\mathrm{AG}}$ and ${\mathrm{LS}}_{\mathrm{BG}}$ are then multiplied by the corresponding fractional decomposition rates ($k_{\mathrm{AG}}\;$and $k_{\mathrm{BG}}$) to calculate $D_{\mathrm{AG}}\;$and $D_{\mathrm{BG}}$ (Equations 15 and 16):

(15) $$D_{\mathrm{AG}}\left(t\right)={\mathrm{LS}}_{\mathrm{AG}}\left(t\right)\times k_{\mathrm{AG}}\left(t\right)$$

(16) $$D_{\mathrm{BG}}\left(t\right)={\mathrm{LS}}_{\mathrm{BG}}\left(t\right)\times k_{\mathrm{BG}}\left(t\right)$$

where the unit of ${\mathrm{LS}}_{\mathrm{AG}}$ and ${\mathrm{LS}}_{\mathrm{BG}}$ is g m^−2^, and that of $k_{\mathrm{AG}}$ and $k_{\mathrm{BG}}$ is day^−1^. We fitted parametric equations with the experimental data using the least‐squares regression technique to estimate $k_{\mathrm{AG}}$ and $k_{\mathrm{BG}}$ as a function of porewater salinity (sal) for different inundation and exposed conditions (Equations 17 and 18). For each iteration, the system compares PE with corresponding WL to determine whether the soil surface was submerged, exposed, or extended exposed to execute the corresponding equations. The change in WL at time tis defined as a function of the WL at time t−1 and SLR (rate of inundation/ponding) as shown in Equation (19):

(17) $$k_{\mathrm{AG}}\left(t\right)=\left\{\begin{array}{c} A_{1}{\left[\mathrm{sal}\left(t\right)\right]}^{2}+B_{1}\left[\mathrm{sal}\left(t\right)\right]+C_{1},\\ \;\text{when}\;\mathrm{WL}\;\left(t\right)\geq \mathrm{PE}\left(t\right)\\ A_{2}+B_{2}\times \ln\left[\mathrm{sal}\left(t\right)\right],\\ \;\text{when}\;\mathrm{WL}\;\left(t\right)-\mathrm{PE}\left(t\right)<0\;\text{and}\\ \;\mathrm{WL}\;\left(t\right)-\mathrm{PE}\left(t\right)\geq -5\;\mathrm{cm}\\ A_{3}+B_{3}\times \ln\left[\mathrm{sal}\left(t\right)\right],\\ \;\text{when}\;\mathrm{WL}\;\left(t\right)-\mathrm{PE}\left(t\right)<0\;\text{and}\\ \;\mathrm{WL}\;\left(t\right)-\mathrm{PE}\left(t\right)<-5\;\mathrm{cm} \end{array}\right.$$

(18) $$k_{\mathrm{BG}}\left(t\right)=\left\{\begin{array}{c} P_{1}{\left[\mathrm{sal}\left(t\right)\right]}^{2}+Q_{1}\left[\mathrm{sal}\left(t\right)\right]+R_{1},\\ \;\text{when}\;\mathrm{WL}\;\left(t\right)\geq \mathrm{PE}\left(t\right)\\ P_{2}+Q_{2}\times \ln\left[\mathrm{sal}\left(t\right)\right],\\ \;\text{when}\;\mathrm{WL}\;\left(t\right)-\mathrm{PE}\left(t\right)<0\;\text{and}\\ \;\mathrm{WL}\;\left(t\right)-\mathrm{PE}\left(t\right)\geq -5\;\mathrm{cm}\\ P_{3}+Q_{3}\times \ln\left[\mathrm{sal}\left(t\right)\right],\\ \;\text{when}\;\mathrm{WL}\;\left(t\right)-\mathrm{PE}\left(t\right)<0\;\text{and}\\ \;\mathrm{WL}\;\left(t\right)-\mathrm{PE}\left(t\right)<-5\;\mathrm{cm} \end{array}\right.$$

(19) $$\mathrm{WL}\left(t\right)=\mathrm{WL}\left(t-1\right)+\mathrm{SLR}\left(t\right)$$

where A,B, *C*, *P*, *Q*, and *R* are model parameters. The unit of WL is cm NAVD 88 and SLR is in cm day^−1^. Water depth (WD) relative to the peat surface (cm) is approximated from the difference between WL and PE (Equation 20). ${\mathrm{LP}}_{\mathrm{AG}}$ and ${\mathrm{LP}}_{\mathrm{BG}}$ in g m^−2^ day^−1^ are approximated from the corresponding ANPP and BNPP as a function of their respective turnover rates (${\mathrm{TR}}_{\mathrm{AG}}$ and ${\mathrm{TR}}_{\mathrm{BG}})$ (Equations 21 and 22):

(20) $$\mathrm{WD}\left(t\right)=\mathrm{WL}\left(t\right)-\mathrm{PE}\left(t\right)$$

(21) $${\mathrm{LP}}_{\mathrm{AG}}\left(t\right)=\text{ANPP}\;\left(t\right)\times {\mathrm{TR}}_{\mathrm{AG}}$$

(22) $${\mathrm{LP}}_{\mathrm{BG}}\left(t\right)=\text{BNPP}\;\left(t\right)\times {\mathrm{TR}}_{\mathrm{BG}}$$

Similar to the estimation of litter breakdown rates, the last term in Equation (8), $∆S_{T}\;$is calculated as a direct function of salinity for different inundation conditions (Equation 23) using the experimental observations as follows:

(23) $$∆S_{T}\left(t\right)=\left\{\begin{array}{c} X_{1}+Y_{1}\times \ln\left[\mathrm{sal}\left(t\right)\right],\\ \;\text{when}\;\mathrm{WL}\;\left(t\right)\geq \mathrm{PE}\left(t\right)\\ X_{2}+Y_{2}\times \ln\left[\mathrm{sal}\left(t\right)\right],\\ \;\text{when}\;\mathrm{WL}\;\left(t\right)-\mathrm{PE}\left(t\right)<0\;\text{and}\\ \;\mathrm{WL}\;\left(t\right)-\mathrm{PE}\left(t\right)\geq -5\;\mathrm{cm}\\ X_{3}+Y_{3}\times \mathrm{sal}\left(t\right),\\ \;\text{when}\;\mathrm{WL}\;\left(t\right)-\mathrm{PE}\left(t\right)<0\;\text{and}\\ \;\mathrm{WL}\;\left(t\right)-\mathrm{PE}\left(t\right)<-5\;\mathrm{cm} \end{array}\right.$$

where XandY are model parameters.

Finally, NECB is computed by balancing the C in the model following Equation (24):

(24) $$\text{NECB}\left(t\right)=\text{ANPP}\left(t\right)+\text{BNPP}\left(t\right)-D_{\mathrm{AG}}\left(t\right)-D_{\mathrm{BG}}\left(t\right)-∆S_{C}\left(t\right)-F_{\mathrm{CH}4}\left(t\right)$$

where the units is gC m^−2^ day^−1^. $\text{ANPP}\left(t\right)$, $\text{BNPP}\left(t\right)$, and $F_{\mathrm{CH}4}\left(t\right)$ are model inputs, and $D_{\mathrm{AG}}\left(t\right)$ and $D_{\mathrm{BG}}\left(t\right)$ are estimated using Equations (15) and (16), respectively. $∆S_{C}\left(t\right)$ is determined from the model estimated $∆S_{T}\left(t\right)$ by adjusting for the fraction of C in soil matter, $f_{C}$, that varies between 0 and 1 (Equation 25):

(25) $$∆S_{C}\left(t\right)=∆S_{T}\left(t\right)\times f_{C}$$

### Implementation of modeling framework in Stella

**FIGURE 3:**
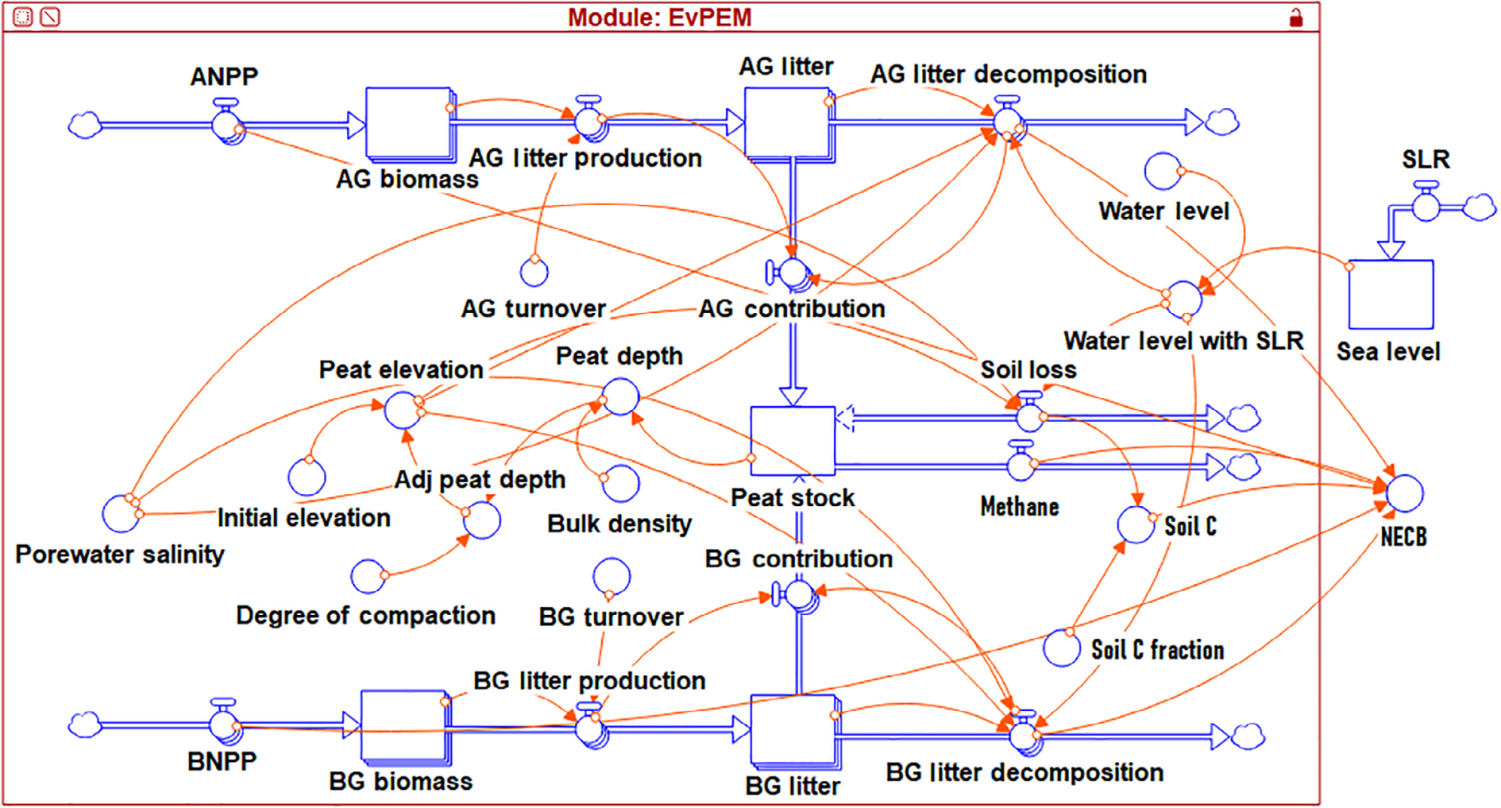
Diagram of system dynamics Everglades peat elevation model (EvPEM) used to simulate change in peat stock ($M_{S}$) and peat elevation (PE). Adj, AG, ANPP, BG, BNPP, NECB, and SLR refer to adjusted, aboveground, aboveground net primary productivity, belowground, belowground net primary productivity, net ecosystem carbon balance, and sea‐level rise, respectively.

We employed the software package Stella version 1.9.2 to implement the modeling framework termed Everglades peat elevation model (EvPEM; Figure 3). Stella is a user‐friendly, flexible tool that offers robust simulations of the framework through the incorporation of different stocks, flows, connectors, and converters (Feng et al., 2013; Appendix S1: Section S1, Figure S2). The model was developed with six stocks and 11 internal and external flows to store, transfer, and quantify inflows and outflows. The required inputs to simulate the model are listed in Table 1. The simulation time unit is in days, and a fractional delta time (DT) is set at 0.125 to run Stella; DT specifies how many times per the unit time the model's numerical values are recalculated. All the inputs of the model were linearly downscaled from year to day to facilitate the daily time step. In each iteration, the Stella model determines the inundation level of the system by simultaneously comparing the PE and WL specified in the modeling framework. The WL is also linked to the option of including SLR (or rate of increase in inundation), a separate sea‐level stock connected with the WL converter to adjust to sea‐level change.

**TABLE 1:** Input variables, types of Stella building block, and units required to simulate change in peat stock ($M_{S}$) and elevation (PE) using EvPEM.

Input variable	Stella building blocks	Unit
Aboveground net primary productivity (ANPP)	Flow	gC m^−2^ day^−1^
Belowground net primary productivity (BNPP)	Flow	gC m^−2^ day^−1^
Methane flux ($F_{\mathrm{CH}4}$)	Flow	gC m^−2^ day^−1^
Initial aboveground litter stock (${\mathrm{LS}}_{AG_{0}}$)	Stock	gC m^−2^
Initial belowground litter stock (${\mathrm{LS}}_{BG_{0}}$)	Stock	gC m^−2^
Aboveground turnover rate (${\mathrm{TR}}_{\mathrm{AG}})$	Converter	day^−1^
Belowground turnover rate (${\mathrm{TR}}_{\mathrm{BG}})$	Converter	day^−1^
Initial peat elevation (${\mathrm{PE}}_{0})$	Converter	cm NAVD88
Initial peat stock	Stock	g m^−2^
Soil bulk density ($\rho_{b}$)	Converter	g m^−3^
Degree of compaction (α)	Converter	unitless
Soil C fraction ($f_{c}$)	Converter	unitless
Porewater salinity (sal)	Converter	ppt day^−1^
Water level (WL)	Converter	cm NAVD88 day^−1^
Sea‐lever rise (SLR)	Flow	cm day^−1^

*Note*: Initial peat stock is the product of initial peat depth and soil bulk density.

Upon parameterizing the model using measured data, we simulated EvPEM for a single year using treatment‐specific data. We upscaled the daily model‐predicted PE change to the yearly scale for each treatment for calibration.

### Statistical analyses and NECB estimates from measured data

Synthesizing the experimental measurements, we analyzed the variation in ANPP and BNPP with increasing salinity for different inundation levels and parameterized them using nonlinear equations. We used one‐way analysis of variance (ANOVA) to compare the differences among the experiments (between salinity treatments and among inundation treatments) for ANPP, BNPP, ${\mathrm{TR}}_{\mathrm{AG}}$, and ${\mathrm{TR}}_{\mathrm{BG}}$ at a 95% level of confidence using measured data. We calculated the treatment‐specific means of all the components listed in Equation (2) with measured data to estimate corresponding NECB and $F_{\mathrm{AQ}}$. We also statistically compared the mean change in $k_{\mathrm{AG}}$, $k_{\mathrm{BG}}$, $∆S_{T}$, $∆S_{c}$, $F_{R}$, and NECB due to the salinity and inundation manipulations.

### Model parameterization and evaluation

Upon setting up the EvPEM in Stella (Figure 3), we parameterized Equations (17), (18), and (23) using least‐squares regression for the SUB treatments across the salinity levels as a function of salinity by combining all the treatment‐specific freshwater and brackish water data. However, because of the unavailability of freshwater drought/exposed treatment data, we parameterized Equations (17), (18), and (23) only using two brackish water AMB and SALT salinity levels for EXP and EXTEXP treatments. We used measured $k_{\mathrm{AG}}$, $k_{\mathrm{BG}}$, and $∆S_{T}$ ($∆S_{T}$ is computed from the observed change in peat depth using Equation 7) for the parameterization. We linearly converted their time dimensions from yearly to daily before parameterizing the model at the daily scale.

We adopted the functional forms that provided the best model fits and minimum uncertainty under each hydrologic treatment. We fitted $k_{\mathrm{AG}}$ and $k_{\mathrm{BG}}$ with a polynomial function for the SUB condition; however, we used the semi‐logarithmic function to fit $k_{\mathrm{AG}}$ and $k_{\mathrm{BG}}$ for the EXP and EXTEXP experiments. Similarly, we parameterized a semi‐logarithmic equation for SUB and EXP and a linear equation for EXTEXP to derive the mathematical expressions for $∆S_{T}$.

Finally, after implementing the model equations in Stella, we simulated and calibrated EvPEM for each treatment where we compared the model‐estimated elevation change and NECB with the observations. We set the initial elevation at zero during calibration and assumed 1.5 m of peat soil layer underlying a limestone bedrock during model implementation. We used observed/measured treatment‐specific ANPP, BNPP, $F_{\mathrm{CH}4}$, salinity, WL, ${\mathrm{TR}}_{\mathrm{AG}}$, ${\mathrm{TR}}_{\mathrm{BG}}$, $\rho_{b}$, and α as inputs for each of the baseline treatment simulations. We obtained the model‐simulated elevations after 1 year of model run as elevation change over a year to compare with the measured PE change. The model calculates the daily simulated NECB (gC m^−2^ day^−1^) for each treatment, where $D_{\mathrm{AG}}$, $D_{\mathrm{BG},}\;$and $∆S_{C}$ ($∆S_{C}$ was calculated from simulated $∆S_{T}$ by adjusting for a fraction of C in the soil mass) were simulated, and ANPP, BNPP, and $F_{\mathrm{CH}4}$ were model inputs (Equation 24). We computed the annual sums (gC m^−2^ year^−1^) from the daily simulated NECB values to compare with the observations. We estimated the standard errors of the model predictions by simulating the model with the standard errors of the estimated model parameters.

We used Nash‐Sutcliffe efficiency (NSE) and mean bias error (MBE) indexes to evaluate the EvPEM simulations (Equations 26 and 27). We combined all seven treatment (*n* = 7) simulations for a combined overall model evaluation:

(26) $$\mathrm{NSE}=1-\left(\frac{\sum_{i=1}^{n}{\left(y_{i,\mathrm{obs}}-y_{i,\mathrm{sim}}\right)}^{2}}{\sum_{i=1}^{n}{\left(y_{i,\mathrm{obs}}-y_{\text{mean},\text{obs}}\right)}^{2}}\right)$$

(27) $$\mathrm{MBE}=\frac{\sum_{i=1}^{n}(y_{i,\mathrm{obs}}-y_{i,\mathrm{sim})}}{n}$$

### Scenario simulations with EvPEM

Based on the EvPEM model simulations, we optimized marsh PE change to develop threshold levels of annual NPP (sum of annual net aboveground and belowground productivity) as a function of increasing salinity (1–20 ppt) and inundation (3 mm year^−1^) for accumulating (stable), steady‐state (no change), and collapsing peats. We ran three example scenarios to determine the annual NPP necessary to represent the (1) accumulating, (2) steady‐state, and (3) collapsing peat by varying NPP, salinity, and WL (Appendix S1: Table S1). The scenarios were simulated for 30 years with a daily time step.

For the stable scenario simulation, NPP was changed from ~130 to ~440 gC m^−2^ year^−1^ over the 30 years with a corresponding salinity change from 1 to 20 ppt. Similarly, NPP was varied from ~160 to ~1070 gC m^−2^ year^−1^ for the 1–20 ppt salinity gradient to simulate an accumulating peat condition. For the collapsing peat, we assumed a declining NPP from ~140 to 40 gC m^−2^ year^−1^ over the simulated period. The WL for the first year of simulation was set to represent the seasonal hydropattern—with 8 months of complete submergence, 3 months of moderate, and 1 month of high exposure of the peat surface. We then imposed a 3 mm year^−1^ (a proxy for mean global rate of SLR; Chen et al., 2017) increase rate in WL over the 30‐year simulation to represent the impact of SLR. We further assumed ${\mathrm{TR}}_{\mathrm{AG}}$ = 1.5 year^−1^, ${\mathrm{TR}}_{\mathrm{BG}}$ = 0.5 year^−1^, $\rho_{b}\;$= 0.13 g cm^−3^, α = 0.1, $F_{\mathrm{CH}4}$ = 0, and initial PE = 0 cm NAVD88 to simulate the scenarios.

## RESULTS

### Aboveground and belowground productivity

**FIGURE 4:**
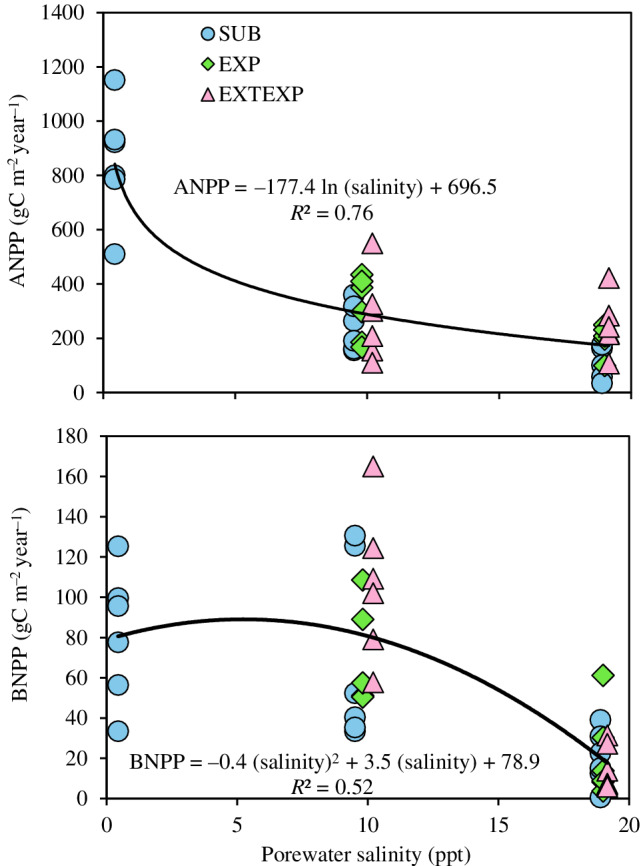
Decline in sawgrass (*Cladium jamaicense*) aboveground net primary productivity (ANPP) and belowground net primary productivity (BNPP) with increasing porewater salinity for submerged (SUB), exposed (EXP), and extended depth of exposure of peat surface (EXTEXP) hydrologic treatments.

The experimental measurements showed that ANPP decreased with salinity across inundation levels (Figure 4). BNPP decreased substantially with the increase of salinity from 10 to 20 ppt but increased marginally with the increase in salinity from 0.5 to 10 ppt. However, combined across inundation levels, mean ANPP (*F*_2,37_ = 60.95, *p* < 0.001) and BNPP (*F*_2,37_ = 21.94, *p* < 0.001) were significantly lower with increased salinity. Furthermore, when ANPP and BNPP were compared across inundation levels for the three salinity levels, the null hypothesis of no significant differences in ANPP (*F*_2,37_ = 1.86, *p* > 0.05) or BNPP (*F*_2,37_ = 0.48, *p* > 0.05) could not be rejected. Therefore, we expressed ANPP and BNPP as a sole function of salinity relative to the hydrologic conditions of the experiments employed. Nonlinear logarithmic and polynomial equations respectively best represented the variation in ANPP (NSE = 0.76) and BNPP (NSE = 0.52) as a function of salinity (Equations 28 and 29):

(28) $$\text{ANPP}=696.5-177.4\;\ln\left(\mathrm{sal}\right)\;\left(F_{39}=122.1,p<0.001,R^{2}=0.76\right)$$

(29) $$\text{BNPP}=78.9+3.5\left(\mathrm{sal}\right)-0.4{\left(\mathrm{sal}\right)}^{2}\left(F_{39}=21.4,p<0.001,R^{2}=0.52\right)\;$$

### Turnover and decomposition rates

Leaf turnover rate (${\mathrm{TR}}_{\mathrm{AG}})$ was higher in the freshwater marsh compared to the brackish water marsh. However, ${\mathrm{TR}}_{\mathrm{AG}}\;$did not vary significantly across salinity (*F*_1,4_ = 0.50, *p* = 0.52) or inundation levels (*F*_2,3_ = 2.90, *p* = 0.20) in the brackish water treatments. Leaf turnover rate declined from the AMB to SALT treatment for submerged (23%) and exposed inundation levels (40%) (Table 2). We found a significant (*F*_2,3_ = 17.95, *p* = 0.02) difference in root turnover rate (${\mathrm{TR}}_{\mathrm{BG}}$) among inundation levels in the brackish water experiment, while ${\mathrm{TR}}_{\mathrm{BG}}$ did not vary (*F*_1,4_ = 0.33, *p* = 0.60) between salinity levels. In particular, ${\mathrm{TR}}_{\mathrm{BG}}$ was higher under EXTEXP (1.3–1.5 year^−1^) compared to SUB (0.54–0.28 year^−1^) and EXP (0.56–0.25 year^−1^) for AMB and SALT treatments, whereas we computed very low ${\mathrm{TR}}_{\mathrm{BG}}$ (0.2 year^−1^) in freshwater treatment.

**TABLE 2:** Average ± 1 standard error of sawgrass (*Cladium jamaicense*) aboveground turnover (${\mathrm{TR}}_{\mathrm{AG}})$ and fractional decomposition ($k_{\mathrm{AG}}$) rates, and belowground turnover (${\mathrm{TR}}_{\mathrm{BG}})$ and fractional decomposition ($k_{\mathrm{BG}}$) rates for the seven hydrologic and salinity treatments representing freshwater and brackish water marshes.

Rate (year^−1^)	Freshwater	Brackish water					
	AMB.SUB	AMB.SUB	SALT.SUB	AMB.EXP	SALT.EXP	AMB.EXTEXP	SALT.EXTEXP
${\mathrm{TR}}_{\mathrm{AG}}$	2.89 ± 2.1	1.08 ± 0.6	0.83 ± 0.7	1.46 ± 0.8	0.88 ± 1.2	1.50 ± 1.8	1.68 ± 2.5
${\mathrm{TR}}_{\mathrm{BG}}$	0.20 ± 0.2	0.54 ± 1.4^a^	0.28 ± 0.7^a^	0.56 ± 0.6^a^	0.25 ± 0.5^a^	1.54 ± 0.8^a^	1.29 ± 0.5^a^
$k_{\mathrm{AG}}$	0.41 ± 0.01	0.38 ± 0.05^a^	0.53 ± 0.06^a^	0.59 ± 0.07^a^	0.50 ± 0.04^a^	0.14 ± 0.4^a^	0.10 ± 0.02^a^
$k_{\mathrm{BG}}$	0.27 ± 0.02	0.24 ± 0.01	0.25 ± 0.01	0.25 ± 0.01	0.28 ± 0.01	0.85 ± 0.1	3.95 ± 1.6

*Note*: SALT = elevated porewater salinity of ~20 ppt, AMB = ~0.5 ppt for freshwater and ~10 ppt for brackish water. SUB, EXP, and EXTEXP, respectively, refer to submerged, exposed, and extended exposed treatments for different salinity levels. Estimations are based on the data reported in Charles et al. (2019), Wilson et al. (2018), and Wilson (2018). Letter a represents significant difference (*p* < 0.05) in means among the inundation treatments (SUB, EXP, and EXTEXP) for brackish water experiments obtained from one‐way analysis of variance (ANOVA). We found no significant differences in means (*p* > 0.05) between AMB and SALT treatments across inundation manipulation for brackish water experiment.

We found significant variation in leaf decomposition rate ($k_{\mathrm{AG}}$) (*F*_2,3_ = 18.69, *p* = 0.02) among inundation levels in the brackish water experiment, although no mean differences (*F*_1,4_ = 0.01, *p* = 0.97) were observed between the salinity levels. For root decomposition rate ($k_{\mathrm{BG}})$, the rate did not vary across the salinity (*F*_1,4_ = 0.71, *p* = 0.44) or inundation levels (*F*_2,3_ = 1.91, *p* = 0.29), although $k_{\mathrm{BG}}$ was higher under higher salinity in the brackish water marsh under EXP and EXTEXP inundation levels. However, the rate remained relatively similar for the SUB inundation levels. We found a five‐fold higher $k_{\mathrm{BG}}$ for the SALT.EXTEXP than that of the AMB.EXTEXP treatment (Table 2).

### Peat elevation change, degree of compaction, and soil C

The measured changes in PE decreased with inundation level (*F*_2,3_ = 7.19; *p* = 0.01) but did not vary significantly between salinity levels (*F*_1,4_ = 0.07; *p* = 0.80; Table 3, Appendix S1: Table S2). The elevation decline was higher for the brackish water EXP treatments—particularly for the EXTEXP, where the measured elevation loss increased to 3.73–4.37 cm year^−1^ compared to the SUB and EXP treatments. The measured soil $\rho_{b}$ for the freshwater treatment was 0.10 ± 0.01 g cm^−3^ (Wilson et al., 2018), which was 25% higher than the freshwater field density of 0.08 ± 0.03 g cm^−3^, the resulting degree of compaction, α = 0.25. For the brackish water treatments, $\rho_{b}$ did not significantly vary across the varying levels of inundation and salinity (Wilson et al., 2018), although it increased substantially compared to the brackish water field measurement. Therefore, we computed α = 0.67 from the average measured $\rho_{b}$ of 0.22 ± 0.03 g cm^−3^ across brackish water treatment levels and a field $\rho_{b}$ of 0.13 ± 0.01 g cm^−3^ for the brackish water experiment.

**TABLE 3:** Average ± 1 standard error of sawgrass (*Cladium jamaicense*) net ecosystem carbon balance and complete carbon budget of the freshwater (FW) and brackish water (BW) marsh.

Variables	Freshwater	Brackish water					
	AMB.SUB	AMB.SUB	SALT.SUB	AMB.EXP	SALT.EXP	AMB.EXTEXP	SALT.EXTEXP
PE ^a^	1.67 ± 0.58	−0.75 ± 0.22	−0.58 ± 0.26	−0.46 ± 0.28	−1.29 ± 0.24	−3.73 ± 0.35	−4.37 ± 0.78
ANPP ^a^	850 ± 80	242 ± 39	89 ± 33	313 ± 50	164 ± 43	275 ± 65	247 ± 42
BNPP ^a^	81 ± 13	70 ± 19	20 ± 6	68 ± 10	21 ± 9	106 ± 15	15 ± 5
$D_{\mathrm{AG}}$ ^a^	−347 ± 2	−92 ± 2	−47 ± 2	−183 ± 3	−81 ± 2	−27 ± 1	20 ± 1
$D_{\mathrm{BG}}$ ^a^	−22 ± 1	−16 ± 1	−5 ± 1	−17 ± 1	−6 ± 1	−72 ± 9	−65 ± 8
$∆S_{T}$ ^a^	1371 ± 351	−214 ± 86	−197 ± 102	3 ± 108	−466 ± 94	−1501 ± 192	−1790 ± 305
$∆S_{C}$ ^a^	562 ± 144	−92 ± 37	−85 ± 44	1 ± 46	−200 ± 40	−645 ± 83	−770 ± 131
$F_{\mathrm{CH}4}^{a}$	5 ± 2	1.21 ± 0.8	−0.30 ± 0.7	2.31 ± 0.7	1.29 ± 0.8	2.31 ± 0.7	1.29 ± 0.8
NECB	1119 ± 229	111 ± 17	−27 ± 8	180 ± 100	−104 ± 7	−366 ± 15	−594 ± 94
NEE ^a^	−2003 ± 189	32 ± 69	215 ± 26	123 ± 124	401 ± 55	698 ± 127	633 ± 83
$F_{R}$	879 ± 42	−144 ± 87	−187 ± 18	−305 ± 225	−298 ± 63	−334 ± 113	−40 ± 10
$F_{\mathrm{AQ}}$	317 ± 186	−52 ± 50	−103 ± 26	−306 ± 271	−98 ± 23	311 ± 30	729 ± 142

*Note*: SALT = elevated porewater salinity of ~20 ppt for BW, AMB = ~0.5 ppt for FW and ~10 ppt for BW. SUB, EXP, and EXTEXP, respectively, refer to submerged, exposed, and extended exposed treatments for different salinity levels. ANPP, BNPP, $D_{\mathrm{AG},}$ $D_{\mathrm{BG}},∆S_{T},$ $∆S_{C},F_{\mathrm{CH}4},$ NECB, NEE, $F_{R}$, and $F_{\mathrm{AQ}}\;$ represent aboveground net primary productivity, belowground net primary productivity, aboveground decomposition, belowground decomposition, net change in soil material, net change in soil C, methane flux, net ecosystem carbon balance, net ecosystem exchange of CO_2_, net residual flux of C, and net aquatic flux, respectively. Units of PE and $∆S_{T}$ are cm year^−1^ and g m^−2^ year^−1^, respectively, while the remaining C balance components are in gC m^−2^ year^−1^. F_CH4_ for the AMB.EXTEXP and SALT.EXTEXP treatments were filled from the corresponding AMB.EXP and SALT.EXP measurements, respectively. A negative sign in $D_{\mathrm{AG}}$, $D_{\mathrm{BG}},∆S_{T},$ $∆S_{C}$, and NECB refer to net loss from the system, whereas a negative sign in NEE, $F_{R}$, and $F_{\mathrm{AQ}}$ refer to net gain in the system.

^a^ Based on data reported in Charles et al. (2019), Wilson et al. (2018), and Wilson (2018).

Similar to PE, both $∆S_{T}\;$and $∆S_{C}\;$declined considerably with drought ($∆S_{T}\;$ = *F*_2,3_ = 41.25; *p* = 0.01; $∆S_{C}$ = *F*_2,3_ = 38.35; *p* = 0.01) across salinity levels. In particular, the soil C loss was many‐fold higher for the EXTEXP treatments (Table 3). The brackish water marshes lost 1790 ± 305, 466 ± 94, and 197 ± 102 g m^−2^ of $∆S_{T}$ in a year, respectively, for EXTEXP, EXP, and SUB inundation levels under elevated salinity.

### NECB, NEE, *F*_CH4_, and $F_{\mathrm{AQ}}$

The response of NECB to elevated salinity varied by inundation/drought treatments (*F*_2,3_ = 8.80; *p* = 0.05) (Table 3; Appendix S1: Table S2). NECB was positive for brackish water SUB (111 ± 17 gC m^−2^ year^−1^) and EXP (180 ± 100 gC m^−2^ year^−1^) treatments under ambient salinity, although we estimated a considerable amount of net C loss with ambient salinity for the EXTEXP treatment (−366 ± 15 gC m^−2^ year^−1^). In contrast, NECB became negative with the elevated salinity—NECB decreased by 75%, 156%, and 63%, respectively, for SUB, EXP, and EXTEXP inundation levels as salinity increased from 10 to 20 ppt. Further, we estimated a net gain in NECB in the freshwater treatment (1129 ± 229 gC m^−2^ year^−1^). Concerning NEE, the brackish water marsh acted as a net source of C with a substantially higher CO_2_ release in the atmosphere for both AMB and SALT treatments (Table 3). The measured $F_{\mathrm{CH}4}$ was considerably lower for the brackish water SUB and EXP treatments across salinity levels (−0.3 to 2.3 gC m^−2^ year^−1^), while $F_{\mathrm{CH}4}$, representing the freshwater AMB.SUB treatment (5 ± 2 gC m^−2^ year^−1^), was 2–3 times higher than the brackish water measurements. However, since $F_{\mathrm{CH}4}$ was not available for the brackish water AMB.EXTEXP and SALT.EXTEXP treatments, we supplemented the missing data with values from the corresponding EXP treatments to balance the C for the EXTEXP treatments.

In the brackish water experiment, net residual flux ($F_{R}$) varied from −40 to −334 gC m^−2^ year^−1^ across salinity and inundation levels, although they did not significantly vary between salinity treatments and among varying levels of inundation (Appendix S1: Table S2). We estimated net aquatic flux ($F_{\mathrm{AQ}})$ from the algebraic sum of $F_{R}$ and $∆S_{C}$ using Equation (5) (Table 3). The brackish water EXTEXP treatments for two salinity levels were the net export of $F_{\mathrm{AQ}}$ (311 and 729 gC m^−2^ year^−1^), but we estimated the net import of $F_{\mathrm{AQ}}$ (−52 to −306 gC m^−2^ year^−1^) for the SUB and EXP treatments across salinity levels. The estimated $F_{\mathrm{AQ}}$ was positive in the freshwater treatment (317 ± 186 gC m^−2^ year^−1^), which was ~28% of the corresponding estimated NECB.

### EvPEM parameterization and calibration

The fitting efficacy of $∆S_{T}$ was better for the SUB (NSE = 0.62) compared to the EXP (NSE = 0.41) and EXTEXP (NSE = 0.21) experiments (Table 4). Similarly, the NSE of $k_{\mathrm{AG}}$ was higher for the SUB than that of the EXP, but the fitting performance was low in the EXTEXP experiment. The $k_{\mathrm{BG}}$ model reasonably explained the data variability for EXP (NSE = 0.43) and EXTEXP (0.54) compared to the SUB (NSE = 0.15) condition. Because only two to three levels of salinity treatments were used for parameterizations, some of the models were not statistically significant (*p* > 0.05) and not well constrained with higher uncertainty estimates.

**TABLE 4:** Everglades peat elevation model equations fitted as a function of salinity (sal) to estimate factional aboveground ($k_{\mathrm{AG}}$) and belowground ($k_{\mathrm{BG}}$) decomposition rates and net change in peat soil ($∆S_{T}$).

Hydrologic condition	Model equations as a function of salinity	NSE	Statistical significance
Submerge (WD ≥ 0 cm)	$k_{\mathrm{AG}}=5.14\times 10^{-06}{\left(\mathrm{sal}\right)}^{2}-6.14\times 10^{-05}\left(\mathrm{sal}\right)+1.47\times 10^{-03}$	0.33	*F*_2,14_ = 3.4, *p* = 0.06
	$k_{\mathrm{BG}}=1.02\times 10^{-06}{\left(\mathrm{sal}\right)}^{2}-2.36\times 10^{-05}\left(\mathrm{sal}\right)+8.67\times 10^{-04}$	0.15	*F*_2,13_ = 1.1, *p* = 0.35
	$∆S_{T}=6.85\times 10^{-01}\ln\left(\mathrm{sal}\right)-1.10$	0.62	*F*_1,12_ = 19.5, *p* = 0.01
Exposed (WD < 0 and WD ≥ −5 cm)	$k_{\mathrm{AG}}=-9.99\times 10^{-04}\ln\left(\mathrm{sal}\right)+4.87\times 10^{-03}$	0.14	*F*_1,10_ = 1.7, *p* = 0.23
	$k_{\mathrm{BG}}=1.65\times 10^{-04}\ln\left(\mathrm{sal}\right)+3.99\times 10^{-04}$	0.43	*F*_1,10_ = 6.7, *p* = 0.03
	$∆S_{T}=1.40\;\ln\left(\mathrm{sal}\right)-2.83$	0.41	*F*_1,10_ = 7.1, *p* = 0.02
Extended exposed (WD < −5 cm)	$k_{\mathrm{AG}}=-1.75\times 10^{-04}\ln\left(\mathrm{sal}\right)+7.77\times 10^{-04}$	0.08	*F*_1,10_ = 0.8, *p* = 0.40
	$k_{\mathrm{BG}}=2.43\times 10^{-02}\ln\left(\mathrm{sal}\right)-5.41\times 10^{0}$	0.54	*F*_1,10_ = 11.9, *p* = 0.01
	$∆S_{T}=1.09\times 10^{-01}\times \left(\mathrm{sal}\right)+2.81$	0.21	*F*_1,10_ = 2.63, *p* = 0.13

*Note*: $k_{\mathrm{AG}}$ and $k_{\mathrm{BG}}$ are in day^−1^, sal is in ppt, and $∆S_{T}$ is in g m^−2^ day^−1^. NSE refers to Nash‐Sutcliffe efficiency, and WD refers to water depth.

**FIGURE 5:**
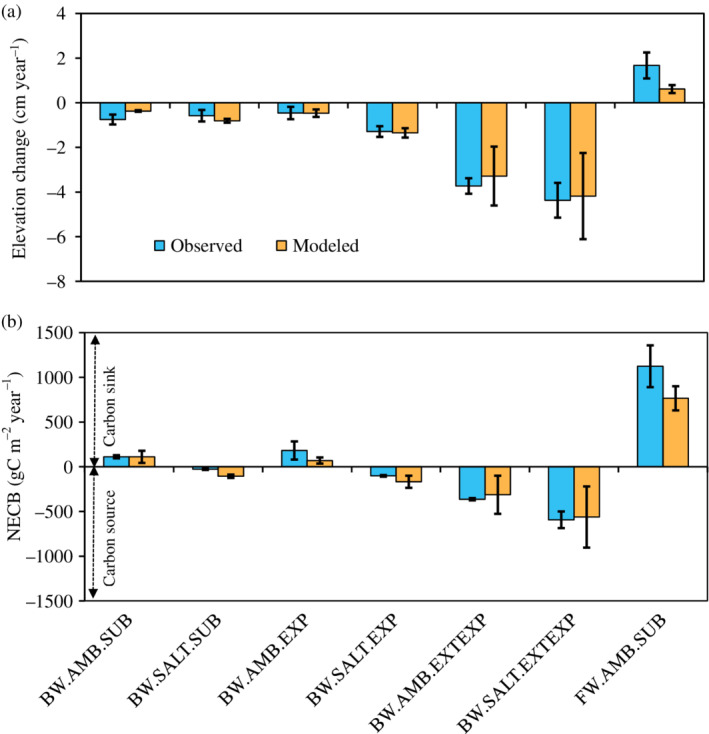
Everglades peat elevation model calibration plot showing comparisons between mean ± 1 standard error experimental observed and simulated changes in (a) peat elevation and (b) net ecosystem carbon balance (NECB) over 1‐year period across the seven treatments representing freshwater (FW) and brackish water (BW) marshes. SALT = elevated porewater salinity of 20 ppt, AMB = 0.5 ppt for FW and 10 ppt for BW. SUB, EXP, and EXTEXP respectively refer to submerged, exposed, and extended exposed hydrologic treatments for different salinity levels. Minus sign refers to loss of elevation and NECB.

The EvPEM calibration showed a good comparison between the observed and simulated mean PE change (NSE = 0.93; MBE = −0.05 cm year^−1^) and NECB (NSE = 0.91; MBE = 76.5 gC m^−2^ year^−1^) combining all the treatments (Figure 5a,b). The simulated elevation changes underestimated the observations within a range of 0.0–1.1 cm year^−1^, where the maximum and minimum deviations in the simulated rates were observed in freshwater AMB and brackish water AMB treatments, respectively. Subject to the brackish water NECB simulations, the prediction error ranged between 50 and 359 gC m^−2^ year^−1^ across the treatments. The standard errors of the predictions were rather large; in particular, we observed higher uncertainty in NECB for the EXP and EXTEXP treatments.

### EvPEM‐based scenario optimization for accumulating, steady‐state, and subsiding peat marsh

**FIGURE 6:**
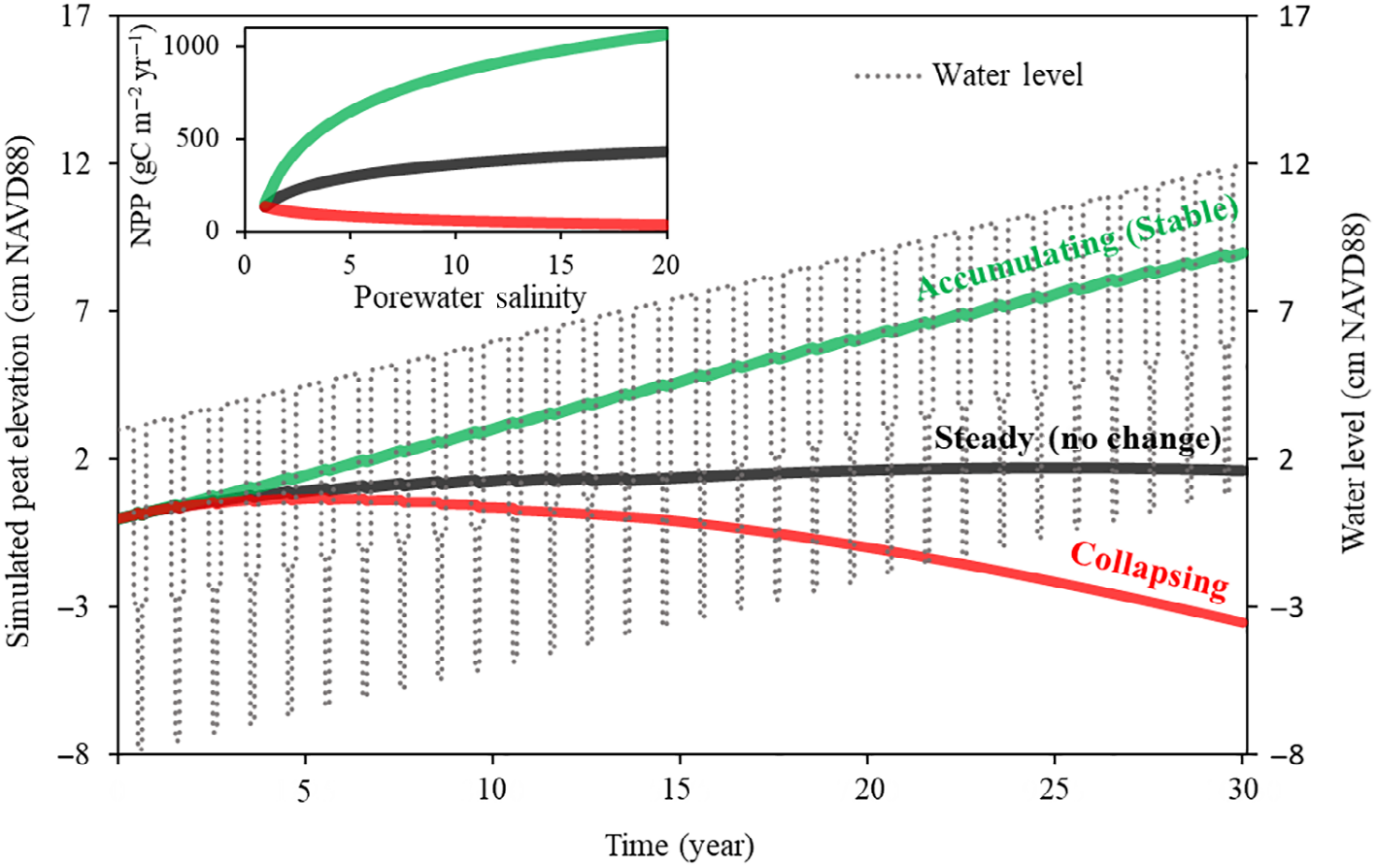
Simulated peat elevations (PEs) in sawgrass marsh under mean global 3 mm year^−1^ sea‐level rise showing examples of accumulating, steady‐state (no change), and collapsing peats in response to annual net primary productivity (NPP), porewater salinity, and hydrology. The inset shows different threshold levels of NPP as a function of salinity used to simulate the corresponding PEs. Ratios of above/below primary productivity, turnover rates, and soil bulk density remained constant during the simulation period. NPP represents the sum of aboveground and belowground net primary productivity. Water level (WL) at the beginning of the simulation (year 1) includes seasonal variability with 8 months of peat soil surface submergence, 3 months of moderately exposed peat soil surface, and 1 month of highly exposed peat soil surface. The WLs of the remaining simulated years were subject to seasonal variability in addition to the overall 3‐mm rise in each year.

The example scenario‐based analysis indicated that ~420–435, 300–420, and 130–300 gC m^−2^ year^−1^ of NPP could reasonably maintain the initial sawgrass PE over time (steady‐state) under lower polyhaline (18–20 ppt), mesohaline (5–18 ppt), and oligohaline (1–5 ppt) conditions, respectively (Figure 6). Under the collapsing scenario, the marsh could potentially drop ~3.5 cm in elevation within 30 years if net productivity declines with increasing salinity. In contrast, the accumulating scenario simulation illustrated the annual NPP required to support peat accumulation (positive elevation change) to avert permanent submergence under continuous salinity exposure. Salinizing marshes would need ~150–550, 550–1030, and 1030–1070 gC m^−2^ year^−1^ of NPP respectively under oligohaline, mesohaline, and lower polyhaline regimes to maintain a constant peat accumulation rate of 3 mm year^−1^.

Because the assumed rate of inundation (3 mm year^−1^) under the steady‐state PE scenario exceeds the simulated rate of PE change (~0.5 mm year^−1^) over the 30 years with an approximate relative inundation rate of 2.5 mm year^−1^, the peat marsh in this scenario would potentially experience a lengthened hydroperiod (from 245 days in year 1 to 325 days in year 30) and would eventually become completely inundated (or close to full ponding) after ~29–30 years. The collapsing scenario simulation indicates a decline in PE at a rate of 1.2 mm year^−1^ as annual NPP decreases with increasing salinity and WL. The relative rate of ponding for the collapsing scenario is nearly 4.2 mm year^−1^, implying the likelihood of an unstable peat system. For example, the model simulation showed that the combined impact of declining productivity and increasing relative ponding for this collapsing scenario would result in total submergence after 21–22 years with a substantially prolonged hydroperiod (33% increase of the number of wet days in a year) within the first 10–12 years of the simulation period.

In contrast, the accumulating PE scenario (Figure 6) represents an elevation growth rate of 3 mm year^−1^ with an assumed increase in annual NPP amid increasing salinity. The growth rate is approximately equal to the assumed mean global rate of SLR of 3 mm year^−1^. Further, the hydroperiod of the accumulating marsh remains unchanged, in contrast to the notable increase in estimated wet days in the steady‐state and collapsing scenarios. Therefore, if the productivity growth profile could be maintained (green line in Figure 6), the accumulating peat would maintain a stable accumulation rate by keeping pace (elevation change ≈ inundation rate) with the increasing level of inundation as the relative inundation/ponding rate approaches ~0 mm year^−1^. However, under field conditions, the simulated optimization scenario could differ as a recent estimate suggests up to 9 mm year^−1^ of SLR in southeast Florida (Wdowinski et al., 2016).

## DISCUSSION

Recent experimental studies (Charles et al., 2019; Servais et al., 2020; Wilson et al., 2018, 2019) helped to better understand the mechanisms of peat collapse in salinizing areas of Florida Everglades. However, little is known about how the overall peat C budget would respond to saltwater intrusion and varying inundation levels and what amount of primary productivity would be necessary to support accumulation of soil C with saltwater intrusion. We developed a system‐level mechanistic modeling framework to estimate and simulate NECB and corresponding peat loss as a function of salinity and WL and applied the model to determine sawgrass productivity necessary to maintain and increase peat accretion under increasing rate of saltwater intrusion. This study elucidated important restoration insights on how reducing salinity through upstream freshwater deliveries could potentially facilitate C accumulation in these collapsing peats by maintaining/increasing the sawgrass primary productivity under accelerated SLR and associated stresses.

### Marsh productivity and decomposition rates

Our analysis indicates that the Everglades sawgrass peat marsh C budget is strongly modulated by saltwater intrusion and dry‐down, which contribute to peat loss. We found a negative trend in ANPP with increasing salinity that varied from freshwater to lower polyhaline regimes (Figure 4); the finding is similar to those of previous studies that reported a decline in sawgrass ANPP with elevated salinity in coastal ecosystems, including the Everglades (Childers et al., 2006; Fuller & Wang, 2014; Lorenz, 2014; Troxler et al., 2014). We did not find a significant influence of submergence or drought on sawgrass productivity. However, the levels of submergence applied to experiments used in this study were low compared with field observations that showed excessive waterlogging and timing of freshwater input impacting aboveground biomass and productivity (Iwaniec et al., 2006; Troxler et al., 2014; Wichern et al., 2006). Despite loss of soil elevation and fine root biomass in salinity treatments, aboveground biomass and NPP of sawgrass were not significantly impacted in freshwater and approximately continuous oligohaline conditions (salinity <7 ppt; Charles et al., 2019); sawgrass can also survive in zones that reach mesohaline conditions (5–18 ppt; Troxler et al., 2014). Long‐term field measurements from the Everglades coastal ecotone show that the number of days of salinity exceeding 30 ppt, discharge, and precipitation can explain 55%–83% of the variation in sawgrass ANPP (Troxler et al., 2014). Based on our application of experimental studies, the polynomial model explained ~76% of the variability of ANPP as a function of salinity alone (Equation 28). The developed models (Equations 28 and 29) can be used to generate ANPP and BNPP profiles along a salinity gradient as inputs for model simulations. However, additional experiments with salinity levels in the range of 0–7 ppt, along with model recalibration, and field validation across a broader range of sites and soil conditions (e.g., marl‐forming marshes and marshes co‐dominated by other plant species) would expand the applicability of the model to different locations across the landscape.

The decrease in leaf and root turnover rates with salinity (Table 2) is consistent with the observed negative correlation between salinity and productivity, contributing to the decrease in soil C stock (Chiang et al., 2000). The decrease in turnover rate would reduce leaf and root litter production, which in turn would have negative feedback on NECB. Subject to the litter decomposition rates, we estimated five‐fold higher root decomposition rates for the EXTEXP treatments, which could be due to the indirect estimation of decomposition rates when compared to the SUB and EXP treatments. However, because water‐holding capacity in highly organic soil is comparatively higher, in general, decomposition rate declines with increasing inundation in peatlands through a positive feedback loop, which in turn contributes to C accumulation (Belyea & Baird, 2006; Ise et al., 2008; Waddington et al., 2015).

### Compaction and soil loss estimation

A sizable fraction of measured elevation loss in the brackish water treatments was attributed to soil compression. Because some areas of the brackish water marsh were already collapsing when we collected the plant–soil monoliths, a higher subsidence rate was expected. Because organic soils have high porosity and low bulk density, coastal peatlands are vulnerable to soil subsidence, autocompaction, and consolidation (Day et al., 2011; Van Asselen et al., 2009), particularly after disturbance (Xiong et al., 2019). The estimated higher compaction for brackish water treatments (α=0.67) is likely due to the initial higher compaction of the already collapsing soil after disturbance, as we extracted the collapsing soil monoliths from the field and moved to the experimental facility. The lower compaction in the freshwater experiment (α=0.25) could be because of the stable soil structure and root system in the absence of salt stress, with the estimated soil accumulation in the freshwater experiment driven by the biomass productivity and CO_2_ uptake (Table 3).

The high oxidation of peats is mainly attributed to the loss of soil C under exposed conditions (Table 3; Appendix S1: Table S2) because C loss is three‐ to four‐fold higher in the EXTEXP treatments. The estimated $∆S_{C}$ from the brackish water peat was ~15% ($f_{C}\times \left(1-\alpha \right)\times 100$) of the total measured elevation loss. Multiplying estimated $∆S_{C}$ by the conversion factor of 3.67, we further calculated the CO_2_‐equivalent of the C loss. We found that extended drought exposure (EXTEXP) resulted in ~2.5 to 3 times higher potential CO_2_‐equivalent emissions under ambient and elevated salinity conditions (24 ± 3 MgCO_2_ ha^−1^ year^−1^ and 28 ± 5 MgCO_2_ ha^−1^ year^−1^, respectively) than for drained tropical peatland secondary forests (10 MgCO_2_ ha^−1^ year^−1^ emission threshold; Hiraishi et al., 2014; Khasanah & van Noordwijk, 2019).

### C balance and aquatic fluxes in response to salinity and inundation levels

There is a wide range of net C exchanges and flux rates in wetlands due to variation and uncertainties associated with drivers of productivity, ecosystem respiration, methane, and aquatic fluxes across spatial and temporal scales, resulting in high variability in NECB (Lu et al., 2017; Waletzko & Mitsch, 2013; Webb et al., 2019). The estimated NECB in previously reported wetland studies that included tropical and temperate coastal freshwater and brackish water wetlands ranged between −393 and 265 gC m^−2^ year^−1^ (Webb et al., 2019); the reported range of NECB was smaller than our estimated range of −594 to 1119 gC m^−2^ year^−1^ across the brackish water and freshwater treatments. Further, the aforementioned span of estimated NECB from our experiments exceeded the NECB range (457 ± 61 to 1038 ± 88 gC m^−2^ year^−1^) that was reported by Troxler et al. (2013) for marsh and mangrove ecosystems within the Florida Everglades.

The observed variability in estimated NECB across freshwater and brackish water treatments resulted from the corresponding variability in NEE, soil C, and productivity (aboveground and belowground) in response to changes in salinity and inundation levels (Table 3). The measured NEE in the brackish water treatments indicated that the marsh was acting as a net source of CO_2_ because of the coupled effect of higher CO_2_ efflux and reduced photosynthesis due to elevated salinity (Negrao et al., 2017; Herbert et al., 2018; Wilson et al., 2018). In contrast, the C‐pool‐based estimates of NECB indicated that the brackish water marsh under ambient salinity was a sink of C for SUB and EXP treatments that converted to a net source when salinity was doubled. A relatively higher primary productivity contributed to the net C gain under ambient salinity condition. On the contrary, the salinity‐driven decline in ANPP and BNPP led to a net loss of C irrespective of the level of inundation. For example, although the peat surface was moderately exposed (4 cm below the surface) in the brackish water AMB.EXP treatment, we estimated a net gain of C under the ambient salinity condition. However, we found a net loss of C in the brackish water SALT.EXP treatment as the total productivity declined by 71% due to elevated salinity (Table 3). Further, though inputs for primary productivity for the AMB.EXTEXP treatment was approximately equivalent to AMB.SUB and AMB.EXP for the brackish water marsh, high oxidation due to extended exposure contributed to the larger negative NECB for this treatment. Therefore, consistent with our understanding of the sawgrass C dynamics in response to SLR and salinity, the NECB estimates render two important implications: (1) SLR‐driven saltwater intrusion can transform the Everglades sawgrass peat marsh landscape from a net sink to a source of C because of decreasing primary productivity and increasing CO_2_ efflux and (2) drought, combined with water management limitations and human water supply needs, can reduce the number of wet days and exacerbate peat oxidation and net loss of C.

The computed residual flux $F_{R}$, as a balance of NECB, NEE, and $F_{\mathrm{CH}4}$, represents the horizontal flow of C in Everglades marshes. We approximate $F_{R}+\left(-∆S_{C}\right)$ as a proxy of $F_{\mathrm{AQ}}$ assuming an absence of particulate C ($F_{Pc})$ in our experiments. Here, $F_{Pc}$ represents particulate C coming from soot and other nonhydrological processes but does not include particulate C originating from peat collapse. The derived aquatic export in the freshwater AMB.SUB treatment (317 ± 186 gC m^−2^ year^−1^) is slightly lower than the range of export of the Everglades marshes (407 ± 63 to 666 ± 61 gC m^−2^ year^−1^), as reported in Troxler et al. (2013), although our brackish water $F_{\mathrm{AQ}}\;$ estimates (−306 ± 271 to 729 ± 142 gC m^−2^ year^−1^) exceeded the Troxler et al. (2013) reported range. Further, our brackish water $F_{\mathrm{AQ}}\;$estimations indicated a net export of $F_{\mathrm{AQ}}$ for the EXTEXP treatments, though we estimated negative aquatic fluxes for the remaining SUB and EXP treatments (Table 3). As our experimental system could only be an export of aquatic C because the plot design allowed the drainage of liberated mass through a pipe, some of the estimated negative fluxes could be biased by measurement uncertainties. If we consider the lower bound of the standard errors of those treatments that had negative aquatic fluxes, NECB almost balances out NEE with a smaller range of negative $F_{\mathrm{AQ}}$ (−3 to −75 gC m^−2^ year^−1^). Because net ecosystem exchange of CO_2_ (−NEE) in brackish water marshes is moderately correlated with $F_{\mathrm{AQ}}$ in our study (*R* ^2^ = 0.63) and brackish water treatments are a net source of CO_2_, net export of $F_{\mathrm{AQ}}$ can be feasible when −NEE is higher than NECB. Therefore, some of the estimated negative $F_{\mathrm{AQ}}$ values suggest the likelihood of underestimation in the measurement of the loss components in Equation (2) as well as the uncertainty in α and $f_{C}$ estimates. It can be a reasonable assumption that the minimum measurement uncertainty of the loss components could be roughly equal to the aquatic flux offset that we derived from NECB and NEE.

Our C balance and budgeting approach included the major components of organic C exchanges (i.e., exchanges of CO_2_, exchanges of CH_4_, burial/loss of C in soil, fraction of DOC) pertinent to a wetland subsystem described in the integrated C balance estimation method proposed by Hopkinson (2018). However, the method proposed in Hopkinson (2018) included fluxes such as transport of particulate and dissolved C, DOC, POC, and DIC. We represented these aforementioned fluxes as residual and aquatic fluxes for C balance closure in our C budget estimation because of the unavailability of direct measurements. Therefore, the C budgeting complexity, uncertainty, and error range underline the value of direct estimation of aquatic C fluxes through the measurements of DOC, DIC, and POC, along with a cross‐site validation for reasonable assessments of C balance combining aquatic and terrestrial C fluxes (Najjar et al., 2018; Troxler et al., 2013; Windham‐Myers et al., 2018). Overall, the estimated C balance and budget across salinity and inundation levels supplement our understanding of the global‐, regional‐, and local‐scale coastal C budgets (Czapla et al., 2020; Herrmann et al., 2015; Najjar et al., 2018; Regnier et al., 2013; Shen et al., 2019) and contribute to multisite and multiscale C data synthesis efforts (Hales et al., 2008). Furthermore, the aquatic export potentials of the marshes suggest a significant transport of flocculent materials and DIC to estuarine and marine ecotones, which has important relevance for estuarine, oceanic, and global C budgets (Alongi, 2020; Najjar et al., 2018).

### Model caveats and future improvement

We used experimental data representing three salinity (~0.5, 10, and 20 ppt) and three inundation (SUB, EXP, and EXTEXP) levels to parameterize the different components of the modeling framework to simulate elevation change. Although we had all three salinity level data for the SUB hydrologic experiment, ~0.5 ppt salinity representing the freshwater domain was not available for EXP and EXTEXP treatments. Therefore, regression‐based parameterizations involving $k_{\mathrm{AG}},k_{\mathrm{BG}},$and $∆S_{T}$ for EXP and EXTEXP only incorporated ambient (~10 ppt) and elevated (~20 ppt) salinity conditions of brackish water marshes. Further, because of limited levels of salinity thresholds, some of the parameterizations are not statistically robust and warrant further improvement using multilevel and multisite data.

Our EXTEXP experiment represents 6 months of submergence and 6 months of drought ranging from −10 to −20 cm of peat soil exposure (Appendix S1: Figure S1); however, the seasonal dry‐down could vary with space and possibly introduce uncertainty into model simulations. The current version of the model did not incorporate the effect of nutrients (e.g., phosphorous), which might stimulate soil C dynamics, plant growth, and turnover rates. The model cannot simulate a positive feedback effect that might impact the rate of C loss. Our model parameterization also included only a short‐term (1‐year) change in sawgrass peat soil, which could be another source of uncertainty in the model. Because of the unavailability of long‐term field measurements of soil surface elevation change via surface elevation tables from sawgrass‐dominated regions of the Florida Everglades, it was not possible to validate model predictions. Future improvements of the model would involve incorporation of multilevel salinity and hydrologic data to increase the range of parameterized model variables by establishing more data collection sites, inclusion of the effect of nutrient enrichment and vegetation dynamics, sensitivity analysis with the robustly fitted model equations, and multisite comparison‐based model validations.

### Conclusions and applications of EvPEM for coastal management

In this study, we estimated NECB and its components for different salinity and inundation levels using experimental data and completed the total C budget for freshwater and brackish water sawgrass marshes. We found that brackish water sawgrass peats transitioned from a net sink to source of C with elevated salinity and lower duration and level of inundation (e.g., drought), whereas the freshwater marsh accumulated C under ambient salinity and submerged conditions.

The simulated C balance and elevation change using the EvPEM was positively related to productivity and negatively linked with loss components, where salinity and level of inundation dictated the estimation of the loss components. The sensitivity of the aboveground and belowground turnover rates depended on both primary productivity and decomposition rates as their balance would eventually contribute to the C stock. Although the model did not incorporate the direct effect of swelling, a negative α can reflect the swelling effect. The presented cell‐scale modeling approach enables a number of features to support water management decisions. It (1) requires a reasonable set of input variables (Table 1) for simulations, (2) utilizes a user‐friendly modeling environment, (3) incorporates the seasonality in WL, (4) accounts for the degree of compaction in peat soil, and (5) enables flexibility in inputs (constant value or time series).

The EvPEM can be used to simulate peat collapse under different inundation/drought and saltwater intrusion scenarios, providing critical information on salinity and productivity thresholds and water management decision support. Applying the model to scenario simulations, we defined annual NPP (sum of ANPP and BNPP) levels required for a stable peat that can keep pace with the inundation rate. Appropriating the productivity optimization, a series of diagrams can be generated by varying different input variables to show the impacts of inundation and saltwater intrusion on Everglades sawgrass peat marshes (Figure 6). Applying these results, we can then define the freshwater delivery protocols required, based on hydrologic and ecologic controls, which can reduce the porewater salinity to maintain or increase primary productivity in Everglades sawgrass peat marshes. A regulated augmentation in freshwater flow into the Everglades that decreases salinity levels is one of the feasible alternatives to inhibit peat loss as outlined in the comprehensive Everglades restoration plan (NASEM, 2018; Sklar et al., 2005; Stabenau et al., 2011). EvPEM represents a significant advancement that enables the exploration of those limiting freshwater thresholds required to maintain a healthy peat through productivity optimization.

The study highlighted the severe susceptibility of coastal wetlands in the Florida Everglades to elevated salinity and hydrologic alterations. SLR‐driven saltwater intrusion, combined with extended dry‐down and climate change, limits the capacity of the wetlands to accumulate C, leading to accelerated elevation loss. Overall, the EvPEM simulations illustrated the importance of plant productivity for maintaining PE in the face of increasing SLR and can be used to develop important sustainable and comprehensive hydrologic and ecological restoration actions. Further, the proposed modeling framework and primary parameterizations with experimental data representing the Everglades sawgrass marsh ecosystem is a useful tool for simulating NECB and elevation change. Specifically, the tool can be applied to similar freshwater‐fed coastal wetlands to derive hydrologic management alternatives to reduce vulnerability of coastal peat marshes in response to SLR and saltwater intrusion. Moreover, the presented approach and model improves our understanding of the sensitivity of biological feedback on coastal wetland change.

## CONFLICT OF INTEREST

The authors declare no conflict of interest.

## Supporting information


Appendix S1

## Data Availability

Data (Troxler et al., 2022) are available from the Environmental Data Initiative at https://doi.org/10.6073/pasta/4593b0279c730e136acaaf88c13e312a.
