# Supplementary material for: Modeling net ecosystem carbon balance and loss in coastal wetlands exposed to sea‐level rise and saltwater intrusion
Source: Ecol Appl. 2022 Aug 12;32(8):e2702. doi: 10.1002/eap.2702 (PMC10078618; doi:10.1002/eap.2702)
Supplement: Supplementary file 1 — Appendix S1 [file EAP-32-0-s001.pdf]

Appendix S1

Modeling net ecosystem carbon balance and loss in coastal wetlands exposed to sea-level rise and saltwater intrusion

Khandker S. Ishtiaq, Tiffany G. Troxler, Lukas Lamb-Wotton, Benjamin J. Wilson, Sean P. Charles, Stephen E. Davis, John S. Kominoski, David T. Rudnick, and Fred H. Sklar

Ecological Applications

Table S1: List of inputs used to simulate the stable, accumulating, and collapsing peat scenarios using EvPEM

Inputs	Scenario: Stable (no change) Peat	Scenario: Accumulating Peat	Scenario: Collapsing Peat
Simulation period (years)	30	30	30
Simulation time-step	Daily	Daily	Daily
Net primary productivity (sum of aboveground and belowground productivity) (gC m^{-2} yr^{-1})	Increased from initial (year 1) 130 to final (year 30) 440.	Increased from initial (year 1) 160 to final (year 30) 1070.	Decreased from initial (year 1) 140 to final (year 30) 40.
Porewater salinity (ppt)	Increased from initial (year 1) 1 to final (year 30) 20.	Increased from initial (year 1) 1 to final (year 30) 20.	Increased from initial (year 1) 1 to final (year 30) 20.
Initial peat elevation (cm NAVD88)	0	0	0
Initial water level (cm NAVD88)	3	3	3
Initial hydropattern	Eight months of complete submergence, three months of moderate, and one month of high exposure of the peat surface.	Eight months of complete submergence, three months of moderate, and one month of high exposure of the peat surface.	Eight months of complete submergence, three months of moderate, and one month of high exposure of the peat surface.
Rate of inundation (mm yr^{-1})	3	3	3
Aboveground turnover rate (yr^{-1})	1.5	1.5	1.5
Aboveground turnover rate (yr^{-1})	0.5	0.5	0.5
Bulk density (g cm^{-3})	0.13	0.13	0.13
Degree of compaction	0.1	0.1	0.1
Methane flux (gC m^{-2} yr^{-1})	0	0	0

Table S2: Results of one-way ANOVA for different key variables comparing (1) mean differences between salinity treatment (SUB vs SALT) across inundation experiments, and (2) mean differences among inundation experiments (SUB, EXP, EXTEXP) across salinity treatments.

Variable	Between salt treatments (AMB vs. SALT) across inundation manipulations	Among inundation treatments (SUB, EXP, EXTEXP) across salt manipulations
PE	$F_{1,4} = 0.07$; $p = 0.80$	$F_{2,3} = 7.19$; $p = 0.01$
ANPP	$F_{1,4} = 4.83$; $p = 0.07$	$F_{2,3} = 0.64$; $p = 0.58$
BNPP	$F_{1,4} = 25.19$; $p = 0.01$	$F_{2,3} = 0.08$; $p = 0.93$
D_{AG}	$F_{1,4} = 1.11$; $p = 0.35$	$F_{2,3} = 2.85$; $p = 0.20$
D_{BG}	$F_{1,4} = 0.13$; $p = 0.74$	$F_{2,3} = 45.46$; $p = 0.01$
ΔS_T	$F_{1,4} = 0.07$; $p = 0.80$	$F_{2,3} = 41.25$; $p = 0.01$
ΔS_C	$F_{1,4} = 0.08$; $p = 0.80$	$F_{2,3} = 38.35$; $p = 0.01$
$NECB$	$F_{1,4} = 0.62$; $p = 0.47$	$F_{2,3} = 8.80$; $p = 0.05$
NEE	$F_{1,4} = 0.30$; $p = 0.61$	$F_{2,3} = 8.27$; $p = 0.06$
F_R	$F_{1,4} = 0.40$; $p = 0.56$	$F_{2,3} = 0.46$; $p = 0.67$
F_{AQ}	$F_{1,4} = 0.34$; $p = 0.59$	$F_{2,3} = 8.04$; $p = 0.06$

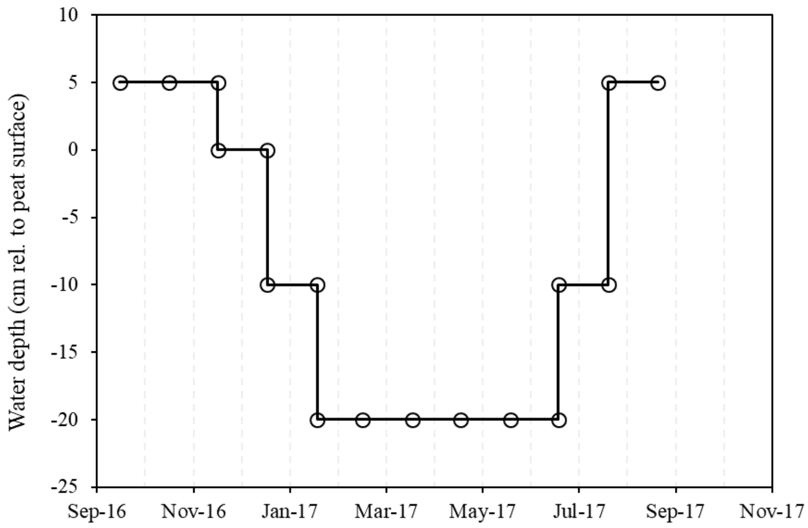

Figure S1: Timing of changes in peat soil exposure (relative to the peat surface) used in the BW exposed with extended seasonal dry-down (EXTEXP) treatments. Water depth profile represents an extended seasonal dry-down of 6 months in the brackish water marsh.

Section S1: Definition of different building blocks of Stella

Stock: Stock represents a building block that accumulates and stores materials. Stock takes materials that flow into, and removes materials that flow out. In this study, a 'reservoir' type stock was used that was represented by a rectangle.

Flow: Flow is a building block that fills or drains stocks. Usually, flow is connected with a stock to provide input materials in the stock or drain out materials from the stock.

Converter: Converter converts inputs to outputs. Holding constant values, defining external inputs to the model, calculating different defined algebraic equations are the key roles of a convertor.

Connector: The connector connects model variables to each other, and represent impact of one variable to another. A connector helps to connect external variables with stock or flows.

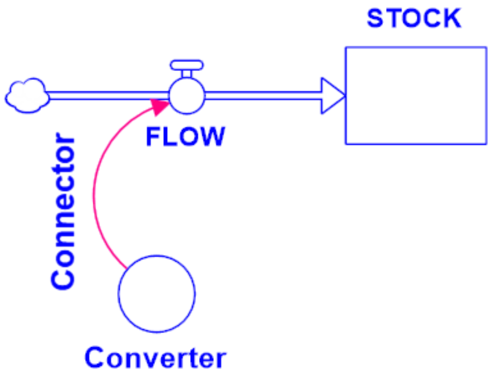

Figure S2**:** A simple representation of major building blocks in Stella

Reference: Stella Documentation, https://www.iseesystems.com/resources/help/v1-9/
